# Pragmatic recommendations to improve access to rehabilitation robots, assistive technologies and neurorehabilitation services in Africa: proceedings from ICORR-SASNET Ghana neurorehabilitation workshop, 2024

**DOI:** 10.3389/fstro.2025.1565651

**Published:** 2025-09-01

**Authors:** Ebenezer Ad Adams, Robert Riener, Mohamed Bouri, Isabel Gunther, Matthew Olaogun, Morenikeji A. Komolafe, Chinonso A. Ad Adams, Albert Akpalu, Mary W. Agoriwo, Leslie W. Ajavon, Kayode Ayodele, Ahmad A. Sanusi, Ahmed O. Idowu, Adebimpe Ogunmodede, Benidict O. Quao, Kang Xiang Khor, Alex Kamadu, Sserunkuma C. Maholo, Shani Halfon, Uchenna C. Eke, Sunday O. Ayenowowon, Emmanuel A. Nelson, Mary C. Barnes, Patience Yeboah, Prince A. Amoah, Charles K. Dakpoe, Mayowa O. Owolabi, Michelle J. Johnson

**Affiliations:** ^1^Stroke Association Supportnetwork-Ghana (SASNET-Ghana), Accra, Ghana; ^2^African Stroke Organization (ASO), Ibadan, Nigeria; ^3^SCI Center, University Hospital Balgrist/University of Zurich, Zurich, Switzerland; ^4^Eidgenössische Technische Hochschule, Zürich (ETH Zurich), Zurich, Switzerland; ^5^Ecole Polytechnique Fédérale de Lausanne (EPFL)/Rehabilitation and Assistive Robotics (REHAssist), Lausanne, Switzerland; ^6^Department of Physiotherapy, Chrisland University, Abeokuta, Ogun, Nigeria; ^7^Department of Medicine, Obafemi Awolowo University, Ile – Ife, Osun, Nigeria; ^8^Department of Physiotherapy, University of Medical Sciences, Ondo, Nigeria; ^9^Department of Medicine and Therapeutics, University of Ghana Medical School, Accra, Ghana; ^10^University of Health and Allied Sciences, Ho, Volta Region, Ghana; ^11^International Rehabilitation Forum/37 Military Hospital, Accra, Ghana; ^12^Department of Electrical and Electronic Engineering, Obafemi Awolowo University, Ile – Ife, Osun, Nigeria; ^13^Department of Medicine, Obafemi Awolowo University Teaching Hospitals Complex, Ile – Ife, Osun, Nigeria; ^14^Family Medicine and Global Health (Infectious Disease) Specialist PM, National Leprosy Elimination Program Ankaful Leprosy General Hospital, Accra, Ghana; ^15^Techcare Innovation, Puchong, Malaysia; ^16^International Society of Wheelchair Professionals, Cape Town, Western Cape, South Africa; ^17^Department of Community and Disability Studies, Kyambogo University, Kampala, Uganda; ^18^TEN-Tikun Olam Empowerment Network, Tel Aviv, Israel; ^19^Department of Physiotherapy, Obafemi Awolowo University Teaching Hospitals Complex, Ile – Ife, Osun, Nigeria; ^20^TEN-Tikun Olam Empowerment Network, Winneba Rehab Project, Winneba, Ghana; ^21^Select Physical Therapy, Franklin, TN, United States; ^22^Department of Bioengineering, University of Pennsylvania, Pennsylvania, PA, United States; ^23^Public Health and Health Promotion Unit, Ministry of Health, Accra, Ghana; ^24^Stroke Association Support Network-Ghana (SASNET Ghana), Accra, Ghana; ^25^Department of Medicine, College of Medicine, and University College Hospital, University of Ibadan, Ibadan, Nigeria; ^26^Center for Genomic and Precision Medicine, College of Medicine, University of Ibadan, Ibadan, Nigeria; ^27^Blossom Specialist Medical Center, Ibadan, Nigeria; ^28^Departments of Physical Medicine and Rehabilitation, Bioengineering and Mechanical Engineering and Applied Sciences, University of Pennsylvania, Pennsylvania, PA, United States; ^29^Rehabilitation Robotics Lab (A GRASP Lab), University of Pennsylvania, Pennsylvania, PA, United States

**Keywords:** stroke, robot-assisted rehabilitation, assistive technology, disabilities, neurorehabilitation, community-based rehabilitation, Africa, low-and middle-income countries

## Abstract

The 2024 ICORR-SASNET Ghana Neurorehabilitation Robotics workshop, convened on March 15-16, 2024, in Accra, Ghana, brought together 22 speakers and 27 attendees from nine countries to address the pressing need for enhanced access to neurorehabilitation services and rehabilitation robotics in Africa. Low- and Middle-Income Countries (LMICs) face substantial challenges in providing adequate rehabilitation services. This exacerbates the burden of disability and impedes the recovery and quality of life of individuals with stroke and other neurological conditions. The workshop aimed to: (1) discuss current trends, challenges in neurorehabilitation services and rehabilitation robotics in Africa; (2) identify gaps in access to rehabilitation services and assistive technologies in LMICs; (3) develop strategies for improving access to these services; and (4) promote collaborative efforts and knowledge sharing among health professionals and stakeholders. A purposive sampling method was employed to recruit a diverse cohort of practicing health professionals, policy makers, and a stroke survivor/advocate. The workshop featured expert presentations and discussions centered on three key questions: (1) the current status of stroke rehabilitation in Africa and driving policies, (2) the role of assistive technology and rehabilitation devices in Africa, and (3) strategies for inclusive implementation culminated into 10 targeted recommendations for integrating rehabilitation robotics into conventional therapies. A roadmap was developed, featuring future initiatives, awareness campaigns, and technology transfer programs, with a planned second workshop in 2026, aiming to enhance access and promote sustainable solutions.

## 1 Introduction

According to the World Health Organization (WHO), 15% of the world's population live with disabilities and 80% of these people reside in Low and Middle-Income Countries (LMICs) (Bright et al., 2018; Mitra et al., 2012). The high disability prevalence in LMICs poses major public health problems due to the expected increase in the demand for assistive technologies and rehabilitation robots as a result of these disabilities. 50% of people with disabilities in LMICs do not have access to the required rehabilitation services due to factors such as: inadequate rehabilitation infrastructure, workforce challenges, limited funding, amongst others (Cyuzuzo et al., 2025). Some of these disabilities are associated with neurological conditions such as stroke. While conventional or traditional stroke rehabilitation, particularly immediately after the acute phase, has been found to improve functional recovery (Duncan et al., 2002), rehabilitation robotics yields better outcomes than conventional training only (Husemann et al., 2007; Krebs and Hamilton, 2024). This is due to its ability to provide more repetitions and hence increase the intensity and dosage of treatment (Krebs and Hamilton, 2024).

In response to the limitations identified in LMICs, International Consortium of Rehabilitation Robotics (ICORR), Stroke Association Supportnetwork-Ghana (SASNET-Ghana) and other stakeholders, organized the 2024 ICORR-SASNET-Ghana neurorehabilitation and robotics workshop under the theme “Community-based neurorehabilitation and robotics in Low- and Middle-Income Countries.”

The workshop was organized to equip rehabilitation professionals with knowledge and skills in neurorehabilitation, assistive technology and rehabilitation robotics. The objectives were to discuss: (1) current trends and challenges in neurorehabilitation services and rehabilitation robotics in Africa; (2) identify gaps in access to rehabilitation services and assistive technologies in LMICs; (3) develop strategies for improving access to these services; and (4) promote collaborative efforts and knowledge sharing among health professionals and stakeholders.

**Figure 1:**
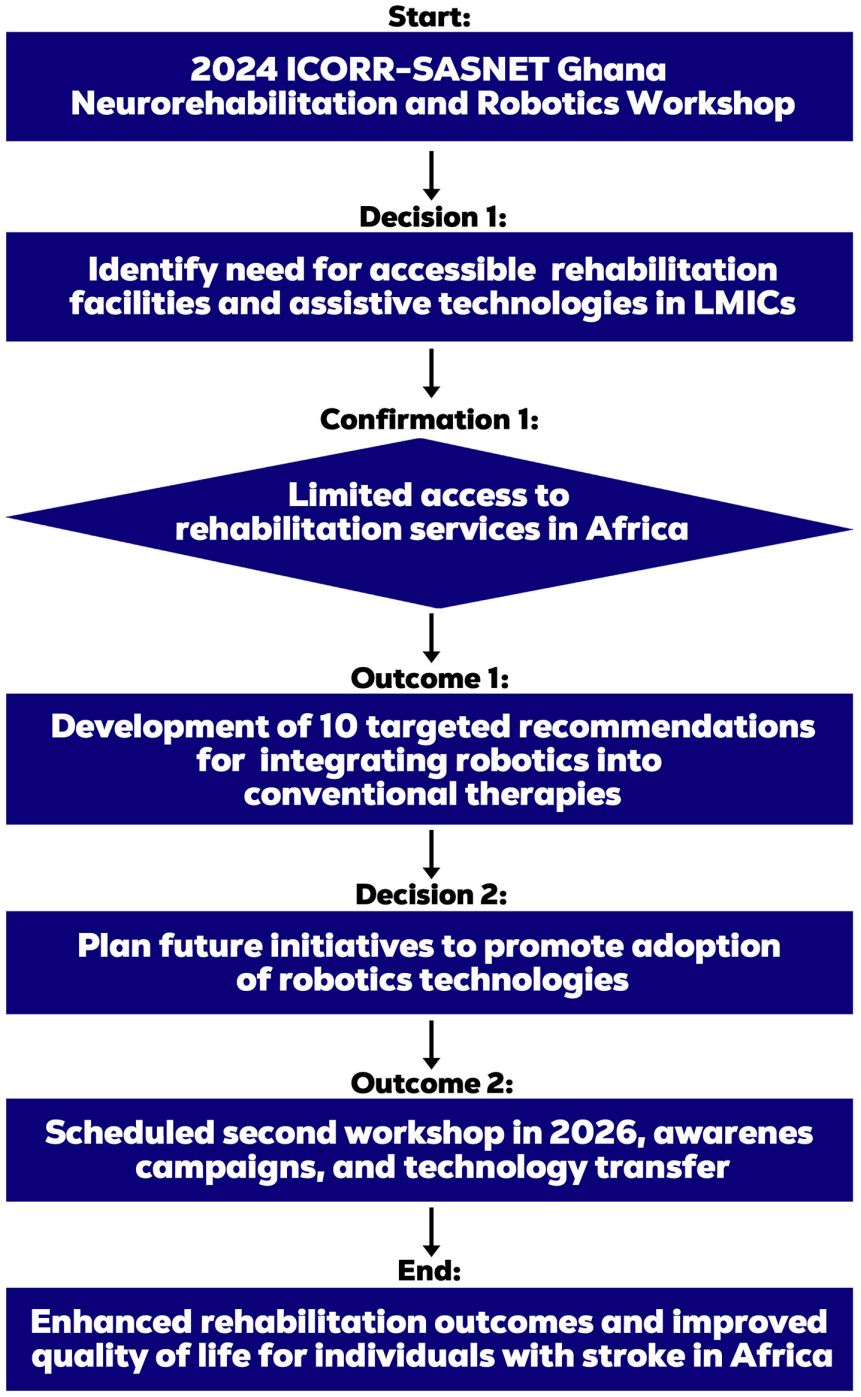
Overview of overarching aims, key discussions and expert cohort composition.

**Figure 2:**
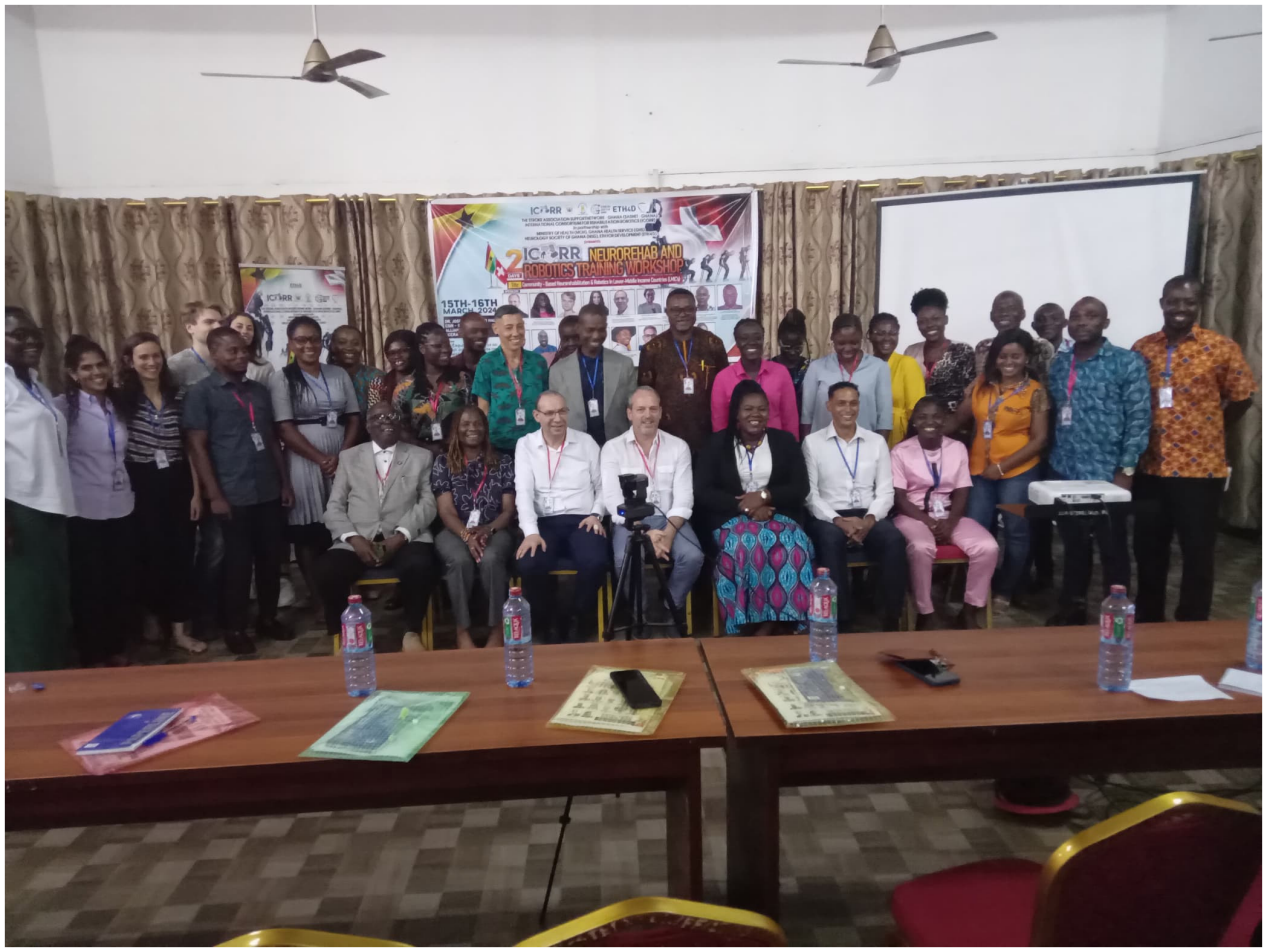
Speakers and participants at the ICORR –SASNET Ghana Neurorehabiliation and Robotics Workshop.

The workshop aims to address several gaps, including limited access to rehabilitation services and assistive technologies, the high costs of rehabilitation robotics and assistive technologies, limited capacity and training for healthcare professionals, limited awareness about the benefits and potential of these technologies, and inadequate policies and frameworks to support their development and implementation in LMICs. Figures 1, 2 provide a visual representation and outline the workshop's framework including its core aims and multidisciplinary expertise.

## 2 Methods

The Neurorehabilitation Robotics Workshop was convened on March 15–16, 2024, at the Center for Scientific and Industrial Research (CSIR) Auditorium. The workshop attendees included a group of participants from the TEN-Tikun Olam Empowerment Network-by the Jewish Agency for Israel, which utilizes a global volunteer model to enhance community resilience in underserved areas through its Health Professions Delegation program. This program brings licensed therapists from Israel to collaborate with local practitioners in a reciprocal learning model, aiming to strengthen rehabilitation services and foster cross-cultural professional development.

The TREAT initiative was integrated into the workshop framework to complement its objective through a multifaceted approach combining Therapies, Research, Enlightenment, Advocacy and Training to enhance the workshop's impact and outcomes. TREAT initiative utilizes an integrated approach including Therapy (multidisciplinary), Research (for cost effective widely applicable, affordable interventions), Enlightenment (to improve public awareness), Advocacy (to mobilize whole society and whole government support), and Training (to produce and sustain the required workforce). This integrated intervention is necessary to produce sustainable and remarkable impact. The workshop featured a multidisciplinary panel of 22 international experts in neurorehabilitation and rehabilitation robotics from nine countries, including Egypt, Ghana, Israel, Malaysia, Nigeria, South Africa, Switzerland, Uganda, and the United States of America. The speaker cohort comprised of engineers, physiotherapists, neurologists, clinicians, and community-based rehabilitation (CBR) professionals.

Participants for the workshop were selected through a purposive sampling method, involving an open call for practicing health professionals with an interest in rehabilitation robotics and a willingness to apply acquired knowledge to improve stroke outcomes. The final cohort consisted of 49 participants, including the 22 speakers and 27 attendees, representing a diverse range of stakeholders such as physiotherapists, stroke nurses, community-based rehabilitation professionals, orthotics and prosthetics, rehabilitation experts, a stroke survivor advocate, and policy makers.

The workshop utilized a structured format featuring expert presentations followed by moderated open discussions to facilitate consensus-building. Through a consensus-driven approach informed by global best practices and expert opinion, the participants generated 10 key recommendations aimed at improving stroke outcomes. Workshop discussions centered on three key questions: (1) the current status of stroke rehabilitation in Africa and driving policies, (2) the role of assistive technology and rehabilitation devices in Africa, and (3) strategies for inclusive implementation of rehabilitation robotic systems in Africa.

## 3 Results

In response to the pressing need for improved access to neurorehabilitation services, assistive technology and rehabilitation robotics in LMICs, the 2024 ICORR workshop brought together international experts and stakeholders to address key questions and objectives.

This section presents the outcomes of the workshop, which aimed to discuss current trends and challenges, identify gaps, and develop strategies for improving access to rehabilitation services and assistive technologies in Africa. Through a consensus-driven approach, the participants generated key recommendations to enhance stroke outcomes and promote community-based neurorehabilitation and robotics in LMICs.

The themes are summarized below.

### 3.1 Capacity building

Stroke rehabilitation aims at attaining patients' maximum independence, physical, mental, social and vocational ability, and full inclusion and participation in all aspects of life (Li et al., 2024). It should start within acute stroke care and must continue throughout the patient's life when necessary. Optimally, a team of specialists are needed, and they are inadequate in the current health system in Africa (Ayalew et al., 2020). To help realize the community-based neurorehabilitation and rehabilitation robotics agenda in LMICs, there is a need to advocate for the training and recruitment of these professionals by the government. Collaboration amongst the available professionals should also be encouraged. Families and caregivers play a very important role in the rehabilitation journey and should also be taught home exercise programmes (Scorrano et al., 2018).

Non-technological strategies to improve neurorehabilitation in Africa were explored. One potential non-technology solution is the use of CBR, which is ideal in the management of many neurological conditions, such as cerebral palsy, stroke amongst others (World Health Organization, 2010; Noukpo et al., 2022). The purpose of CBR is to help individuals improve their functions and wellbeing within their community settings using locally available resources. It may involve a range of stakeholders and therapists depending on patients' condition, the environment in which they live, and their health, psychological, economic and social needs. The barriers to implementing CBR in LMICs include negative attitudes, inadequate training, and gaps in policy, amongst others (Ayalew et al., 2020; Naicker et al., 2019; Hartley and Okune, 2008).

Tikkun Olam Empowerment Network (TEN)'s representative discussed their novel concept of cross-cultural alternative rehabilitation solutions, which encourages the constitution of multidisciplinary and multicultural teams of professionals that collaborate to provide holistic care for patients. It supports community development processes and collaborates with local partners, physiotherapists, teachers of special education and other agents of change in achieving patient's long-term goals. Collaboration across different cultures and disciplines enhances the perspectives of the team, leading to improved outcomes for the patients.

TEN's concept of health professionals' collaboration is currently implemented in Ghana and Uganda only. In Ghana, it operates one volunteering center in Winneba and the health professionals engage in four different settings. Three times in a year, health professionals from Israel visit these centers and spend 5 weeks to work with their local counterparts to provide holistic care for the patients.^1^

Prof. Mayowa Ojo Owolabi discussed the Intersectoral Global Action Plan (IGAP) initiative on epilepsy and other neurological disorders 2022–2031. IGAP was adopted at the 75th World Health Assembly to prioritize neurological disorders, situating brain health at the center of the global health agenda (World Health Organization, 2024). To help improve brain health in LMICs, African countries are encouraged to implement IGAP, focusing on strategies to overcome limitations in the existing services. As such rehabilitation must be integrated into national and subnational health priorities.

A novel concept is the expansion of interdisciplinary care to involve newer relevant fields, including medical humanities to offer holistic care. In Africa, the spiritual sphere to neurological ailments is very important owing to its documented role in the re-establishment of continuity of self along the path to recovery and self-rejuvenation after stroke. Specific approaches that could be explored in this emerging field of neurorehabilitation include motivational, spiritual, energy and arts therapy, amongst others. These approaches require musicologists, religious studies, paranormal science, communication and language artists, amongst others. Therapies, Research, Enlightenment, Advocacy, and Training (TREAT) Agenda must be promoted.

Barriers to implementing the proposed solutions in LMICs include dealing with inadequate rehabilitation facilities and trained therapists and the cultural and social beliefs. To address these challenges, it will be necessary to establish career pathways and training institutions that will produce the required therapists. Also, in LMICs, many individuals are not familiar with rehabilitation and do not demand for it. So, there is a need to improve sensitization at the community level.

### 3.2 Policy recommendations

About 8% of Ghanaians have disability challenges^2^ and require the use of assistive devices to improve their functioning, independence, and wellbeing (World Health Organization, 2010). In the Ghanaian clinical context, access to appropriate assistive devices is recommended for individuals with disabilities but there has been a reported dissatisfaction with the sizing, durability, and associated secondary impairments (De-Rosende-Celeiro et al., 2019; Zuurmong et al., 2018). The appropriateness and safety of the devices should be a priority for the therapists, patients, and caregivers (Tangcharoensathien et al., 2018).

Partnering with qualified engineers, technicians and artisans to design and produce these devices should also be encouraged. Most importantly, government subsidies and policies supporting access to assistive devices in Ghana should be advocated (Kamban and Norman, 2012). For example, there is a huge unmet need for wheelchair ambulation in developing countries, including Ghana, where 90% of people who need wheelchairs do not have access. An estimated 90% of all wheelchairs in Ghana are hand-rim propelled, a physically straining form of ambulation that can lead to repetitive strain injuries and eventually to secondary impairments and disability. This disability can lead to a sedentary lifestyle and thereby create a greater risk for cardiovascular problems.

There is a need for countries to collaborate with organizations such as the International Society of Wheelchair Professionals (ISWP)^3^ and other pertinent organizations (see Table 1); notably, ISWP empowers wheelchair users and their families through training, service, and system-level recommendations, provision of standardized guidelines, and promotion of evidence-based practices. Effective policy development and implementation for assistive technology and stroke rehabilitation require interdisciplinary collaboration between countries and key global organizations.

**Table 1:** Organizations supporting the development and implementation of assistive technologies in stroke rehabilitation.

**Name of organization**	**Mission of organization**	**Contributions**
World Health Organization (WHO)	Offers technical expertise and advocacy to advance assistive technology accessibility and rehabilitation care. Establishes global standards for assistive technology and rehabilitation services. Promotes rehabilitation services and support the integration of rehabilitation into national health systems.	Technical guidance, policy development and implementation assistance.
World Stroke Organization (WSO)	Accelerates advancement in stroke care and prevention through strategic global advocacy, evidence-based education and innovative research initiatives. Promotes optimal stroke outcomes.	Policy development, capacity building /training, awareness/education.
World Federation for Neurorehabilitation (WFNR)	Enhances the field of Neurorehabilitation through scholarly education, research initiatives and global partnership. Promotes a culture of best practices, and continuous quality improvement.	Implementation of program and training/capacity building.
African Stroke Organization (ASO)	A Pan-African organization fostering collaboration among healthcare professional, researchers. Advances stroke prevention, treatment, and rehabilitation outcomes across, through evidence-based research, capacity building, development of stroke services, and policy influence.	Policy development, capacity building/education, technical assistance and implementation of program.
World Heart Federation (WHF)	Advances global cardiovascular health through systemic application of evidence-based interventions, mitigating the impact of cardiovascular diseases and stroke. Promotion of optimal heart health and quality of life for individuals and communities globally.	Policy development, capacity building /research, awareness/advocacy.
Global Partnership for Assistive Technology (ATScale)	Accelerates large-scale implementation and market shaping of assistive technology innovation. Fosters collaboration partnership, research-driven solutions, and strategic market interventions to improve access and outcomes for individuals with disabilities.	Scale up technology transfer and capacity building.
Global Alliance of Assistive Technology Organizations (GAATO)	Supports global coordination and knowledge exchange initiatives, focusing on assistive technology research, development, and implementation. Promotes innovation, capacity building and improved health outcomes worldwide.	Technical assistance, capacity building, development, coordination and implementation of assistive technology policy.
Global Cooperation on Assistive Technology (GATE)	Delivers evidence-based technical expertise and capacity-building initiatives. Advances the Incorporation of assistive technology innovations to augment healthcare system worldwide.	Technical assistance and capacity building.
Community Based Rehabilitation Africa Network (CAN)	Supports the development of robust community-based rehabilitation programs. Promotion of inclusive growth and equitable access to healthcare services across Africa, with a focus on empowering marginalized communities.	Implementation of community- based rehabilitation program and capacity building.

### 3.3 Technology adoption

Robotic devices have evolved over the past 20–30 years from being incredibly heavy and dangerous to safer, intelligent, efficient, and interactive rehabilitation devices (Pollock et al., 2014; Haddadin, 2014). Yet there is a need for these devices to be more lightweight and affordable. In the rehabilitation of patients with stroke, a higher dose of intensive training is required to produce successful outcomes. Evidence from research has shown that robot-assisted therapy gives better results than only conventional training (Duncan et al., 2002; Husemann et al., 2007). Therefore, combining the therapist's experience with the robot's endurance, force and higher doses should be the recommendation needed to bring about successful therapeutic outcomes.

Potential rehabilitation robotic systems such as exoskeleton/exosuits for chronic stroke patients and children with cerebral palsy were reviewed and found to improve walking, speed and endurance (Rodríguez-Fernández et al., 2021; Basla et al., 2024). Also discussed during the workshop was an autonomous wheelchair (Salvini et al., 2022) which can enable people with impairments navigate expansive spaces and complex environments like airports, malls, and museums efficiently and safely without encountering huge risks (Pollock et al., 2014; Sahoo and Choudhury, 2023; Sukerkar et al., 2018).

Robotics is a solution for rehabilitation because it combines elements of mobilization (structures & actuators) and evaluation (sensors). Presently, robot-assisted devices are very expensive high-tech solutions. To reduce costs and make them more affordable in LMICs, there will be a need to increase the production numbers and optimize the design, production, testing, and marketing (Johnson and Mendonca, 2022). The technology could also be transferred to LMICs, where the design and production can be done with local expertise in tandem with report by Lauretti et al. on the development of sustainable robotic solutions in LMICs (Lauretti et al., 2025).

In LMICs, inclusive rehabilitation robotics can help bridge the gap in care caused by shortage of healthcare workers and increasing disabilities from both non-communicable and communicable diseases (Johnson et al., 2024). The goal of this technology is to create rehabilitation robots that can work as smart assistants with clinicians or therapists to evaluate patients' degrees of impairments and provide rehabilitation in challenging environments. Additionally, the technology can provide potential autonomous and semi-autonomous therapy to many populations who may benefit from cognitive and motor rehabilitation.

Despite its hefty price tag, there is acknowledgment that this technology may have certain advantages (Ekechukwu et al., 2020; Yang et al., 2017). Germany, Spain, Switzerland, the United States America, Japan, and South Korea are the wealthy nations primarily involved in the development and marketing of robots. Africa is lagging behind the developed world in rehabilitation robotics, and the possibility exists that relatively few Africans are utilizing this over $2.6 billion market (Kimmatudawage et al., 2024). To be able to compete and benefit from these opportunities, there is a need to develop both the hospital systems and technological engineers in Africa. The high cost of robots, limited resources, lack of infrastructure, and lack of nascent technology are some of the barriers to the advancement of inclusive rehabilitation robotics in Africa.

Table 2 presents 10 strategies for inclusive neurorehabilitation and rehabilitation robotics in order of priority.

**Table 2:** Inclusive neurorehabilitation and rehabilitation robotics strategies.

**Strategy number**	**Strategy description**
**#**	**High priority**
1	Removing barriers to clinical usability and accessibility by developing affordable devices
2	Capacity building and raising awareness for rehabilitation services
3	Addressing the belief systems. Policy concerns and legal frameworks
4	Promoting Community Based Rehabilitation
5	Increasing potential use among patients with complex rehabilitation needs
**#**	**Medium priority**
1	Using low-cost robotics/mechatronic systems or multiple use systems or a system of robot units that can re-configure
2	Developing open-source devices
3	Creating local infrastructure to design and develop these technological solutions
4	Developing social robots
5	Using local manufacturing resources and cheaper materials—3D printing/soft robots/found objects

In some developed countries, such as the United States of America, strategies involving the use of 3D printing have been deployed. Hand and wrist injuries can be safely and effectively managed with the use of 3D-printed assistive devices from plastic materials, which are very affordable compared to anti-claw orthotic, taping, and rubber bands made from thermoplastic materials. Moreover, 3D printers can be used to produce goniometers and grips, making this technology very cost-effective in LMICs, and implementable in community-based neurorehabilitation and rehabilitation robotics programs (Lunsford et al., 2017).

One strategy is to develop simple robots (robotic components) for rehabilitation solutions to help restore and improve patients' motor, sensory and cognitive functions. There is a possibility to develop relevant solutions with simple components in different countries in Africa through research collaboration. Simple rehabilitation robotic strategies, models, approaches, and solutions like the **Lokomat**, and its mechanical impedance approaches—currently developed by the EPFL research group REAssist, the **LegoPress** provided by RehabKits, **Handreha**, a new hand and wrist haptic device for hemiplegic children and exoskeletons **eWAN** and **eWalk** can be used to inspire and promote rehabilitation technology in emerging markets (Bouri et al., 2013; Olivier et al., 2014; Manzoori et al., 2024). Most of these technologies are open source, making their software accessible to people who need them. Johnson and colleagues have implemented **TheraDrive** in Botswana to be an assessment tool for those with motor and cognitive impairment due to stroke and HIV (Johnson et al., 2017; Kebaetse et al., 2024).

Another promising strategy is the use of affordable hand rehabilitation robots, such as exoskeletons capable of delivering more than 20 types of training configurations for hand recovery compression therapy, passive and mirror exercises, active-assisted movements, and resistive exercises. The Techcare Hand Robot (HR-30), introduced by Dr. Khor Kang Xiang from Malaysia, can be tailored to stimulate over 30 types of hand prehension and functional movements.^4^ This allows patients to continue their rehabilitation at home, maintaining training consistency beyond the clinical setting.

Notably, the robot enables significantly more intensive training; up to ten times more than conventional therapy. For example, it can deliver approximately 1,000 repetitions in 60 min (at a pace of one repetition every 3 s maximum), compared to about ~40 repetitions typically achieved in a standard therapy session. Combining robotic therapy with conventional therapeutic exercises is essential to maximize functional recovery. Hand rehabilitation robots play a crucial role in enhancing neuroplasticity through targeted, repetitive, and high-intensity motor training (Calabrò et al., 2018). However, despite being relatively affordable, the cost of these devices remains a significant barrier to widespread adoption and scalability.

A pilot survey was conducted at Ile-Ife, Southwest Nigeria, on the use of the Platform for Upper Limb Stroke Rehabilitation (PULSR) (Komolafe et al., 2024), an end-effector rehabilitation robot introduced in 2022. The survey revealed that despite the potential benefits of human-robot interaction in stroke rehabilitation, more than 58% of respondents were unaware of the benefits of rehabilitation robotics for stroke care.

The major determinants of interest in rehabilitation robotics were mainly the outcome after the first trial in other participants (44%), an increase in awareness and knowledge about robotics (29%), and closer proximity (13%) (World Health Organization, 1978). The major barriers to participating in robotic stroke rehabilitation were lack of financial support and transport aid as well as long distance to access such care whilst the facilitators to participation were creating awareness and the availability of transportation aid from home to hospital.

The key factors responsible for the acceptance of robot-assisted therapy for stroke rehabilitation in Africa included education and awareness, trust in robot-assisted therapy, cultural factors and beliefs, perceived long-term outcomes, access to robot infrastructure, perceived benefits and efficacy, and family support. The study concluded that robotic rehabilitation is feasible in developing countries and offers solution to low manpower but most stroke survivors in Africa are not aware of the usefulness and advantages of rehabilitation robotics in stroke rehabilitation (Roy and Mavuduri, 2020). As such, advocacy for increasing knowledge and creating awareness about rehabilitation robotics was recommended.

Making these technologies more feasible requires partnerships. For example, in Ghana, ETH Zurich has implemented ETH for Development (ETH4D), which supports research, technological innovation, engineering, and collaborations aimed at identifying new solutions to improve the lives of people in LMICs (Gocevska et al., 2024; Tannor et al., 2024). ETH4D trains engineers and natural scientists to develop, implement, and scale up world- changing innovations with a global perspective. Over the years, ETH4D has worked with various African universities on numerous academic exchange programs and dialogues. These opportunities could be harnessed to support the community-based neurorehabilitation and rehabilitation robotics agenda in LMICs (Demofonti et al., 2021).

## 4 Discussion and recommendations

The workshop speakers presented a comprehensive framework for integrating rehabilitation robotics into African rehabilitation settings, emphasizing the potential benefits of these technologies while critically examining the associated challenges and proposing evidence-based solutions to overcome these obstacles.

The availability of rehab robots improves the lives of people living with disabilities, reduces the dependence on caregivers and reduces the cost of traveling to rehab centers. This is due to the portability of some rehabilitation robots, allowing patients to continue their rehabilitation at home with less frequent visits at the rehab centers (Wagner et al., 2011). Some current issues preventing the accessibility of these resources in African countries are due to lack of education on rehabilitation, high cost of these robots, policies that do not favor people with disabilities and no maintenance culture for these devices in terms of technologists or engineers to fix the devices when faulty. Solutions were proffered to these problems.

The findings in this workshop align with previous research on rehabilitation robotics practices in Ghana, Nigeria, Uganda, Botswana, Egypt, South Africa, Sierra Leone, and the Gambia. It demonstrated that neurorehabilitation robotics is feasible in developing countries, corroborating the notion that effective implementation can transcend economic boundaries (Johnson and Mendonca, 2023).

Emerging therapeutic interventions, including energy-based brain therapies and cross-cultural rehabilitation models, have shown promising outcomes in enhancing patient recovery and functional independence. Additionally, assistive technologies such as robotic devices, 3D-printed assistive devices, autonomous wheelchairs, exoskeletons, and exosuits have demonstrated potential in improving mobility, accessibility, and overall quality of life for individuals with disabilities.

CBR approaches have also been effective in promoting inclusive and sustainable rehabilitation practices. These innovations are supported by growing evidence and hold promise for addressing the complex needs of individuals with disabilities from stroke and neurological conditions.

**Figure 3:**
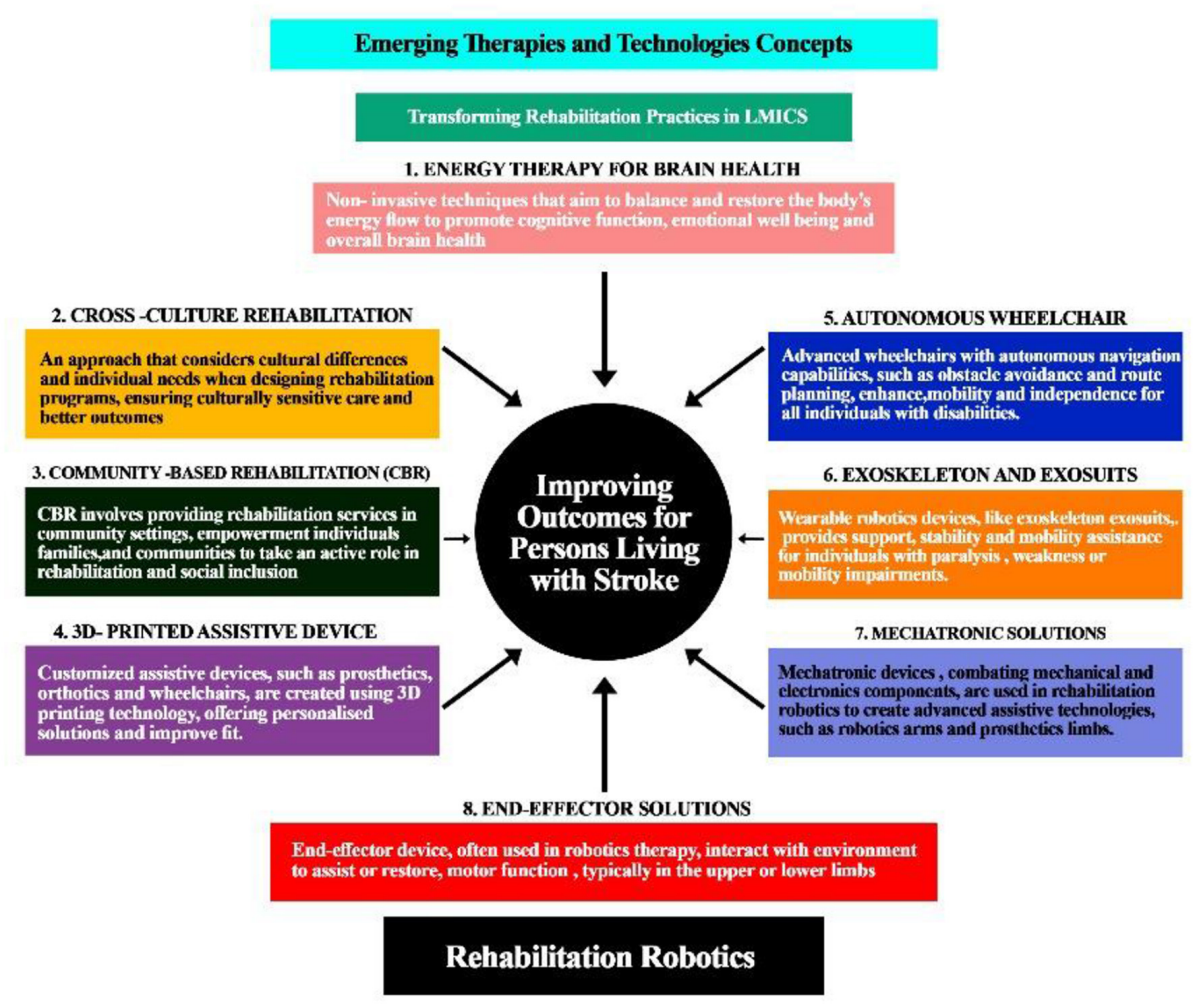
Emerging therapies and technologies are transforming rehabilitation practice and improving outcomes for individuals with disability from stroke.

The integration of innovative therapies and technologies is poised to revolutionize rehabilitation practices in LMICs, enhancing access, personalization, and efficacy of care for individuals with stroke-related disabilities, and ultimately improving functional outcomes and quality of life. These emerging therapies and technologies include energy-based interventions for brain health, cross-cultural rehabilitation approaches, community-based rehabilitation models, 3D-printed assistive devices, autonomous wheelchairs, exoskeletons and exosuits, mechatronic solutions, and end-effector devices as illustrated in the workflow (see Figure 3).

The workshop yielded a comprehensive set of group-level products, including 10 evidence-based recommendations (as detailed in Table 3), policy and practice frameworks, and collaborative initiatives designed to address the multifaceted challenges in neurorehabilitation and rehabilitation robotics. These outcomes have the potential to increase access to essential services and technologies, mitigate existing disparities, and enhance stroke outcomes and quality of life for individuals in LMICs. The consensus-driven recommendations and frameworks generated through this process will provide a foundation for informing future research, policy development, and capacity-building initiatives in LMICs, ultimately contributing to improved healthcare outcomes.

**Table 3:** Recommendations.

**Number**	**Recommendations**
1.	**Foster interdisciplinary collaboration:** Promote the integration of multidisciplinary teams to deliver comprehensive, patient-centered care in both community-based and clinical settings, thereby enhancing holistic rehabilitation outcomes.
2.	**Advance collaboration:** Strengthen stakeholder partnerships, collaborations, and advocacy initiatives to drive inclusive rehabilitation and robotics. Leveraging a transdisciplinary approach that spans the entire innovation value chain, from research and development to product commercialization, to improve the accessibility, efficacy, and affordability of rehabilitation technologies.
3.	**Enhance awareness and policy development:** Promote robot-assisted rehabilitation, assistive technology (AT), and community-based rehabilitation (CBR) through multi-stakeholder awareness campaigns and policy development at national and regional levels by engaging international organizations, government ministries, healthcare professionals, disability organizations, community leaders, private sector entities, civil society organizations (CSOs), academia, and professional organizations.
4.	**Mitigate Cultural and socio-cultural barriers:** Implement community-based initiatives to address cultural, religious, and social determinants influencing the adoption of inclusive rehabilitation and robotics in Africa. This involves conducting targeted outreach and education to promote awareness of robotic rehabilitation benefits, collaborating with local stakeholders to co-design and develop culturally sensitive and economically viable rehabilitation robots, and prioritizing cultural competence in rehabilitation robotics development.
5.	**Facilitate technology transfer and capacity building**: Implement strategic partnerships to transfer robotic technology solutions to African settings. Harnessing local expertise and resources to enhance affordability, accessibility, and cultural relevance by leveraging local capacity, including academic institutions, rehabilitation centers, and regional organizations. These initiatives can accelerate the adoption of rehabilitation robotics, improve neurorehabilitation outcomes, and enhance the quality of life for individuals with disabilities in LMICs.
6.	**Enhance mobility assistive technology:** Develop and implement national wheelchair guidelines, standardize wheelchair prescription protocols, and promote the adoption of smart wheelchairs in African countries, leveraging support from organizations like the International Society of Wheelchair Professionals (ISWP) to inform policy development.
7.	**Capacity building and knowledge transfer:** Establish pre-service and in-service training programs for healthcare professionals in wheelchairs, neurorehabilitation, and robotics solutions, complemented by academic exchange programs between international universities to empower African candidates with advanced knowledge, skills, and global networking opportunities, driving innovation and improving patient outcomes.
8.	**International collaboration and knowledge sharing:** Host the 2nd African Rehabilitation Week workshop in 2026, bringing together experts in neurorehabilitation and robotics from Africa and globally to share knowledge, best practices, and innovations, with potential host countries including Ghana, South Africa, Nigeria, or Botswana.
9.	**Sustained professional engagement:** Establish a core group of professionals who participated in the workshop to facilitate ongoing engagement, training, and information sharing on neurorehabilitation and rehabilitation robotics, ensuring continued momentum and collaboration. This core group will ultimately evolve into a comprehensive Rehabilitation Robotics and Assistive Technology community for Africa, fostering a pan-African network of experts, innovators, and practitioners driving advancements and excellence in the field.
10.	**Hands-on training and innovation:** Establish a Mini-Rehabilitation Robotics Lab in Ghana, providing practical hands-on experience in neurorehabilitation and rehabilitation robotics through partnerships with renowned international institutions, leveraging their expertise, funding, and technical assistance to drive innovation and capacity building.

**Figure 4:**
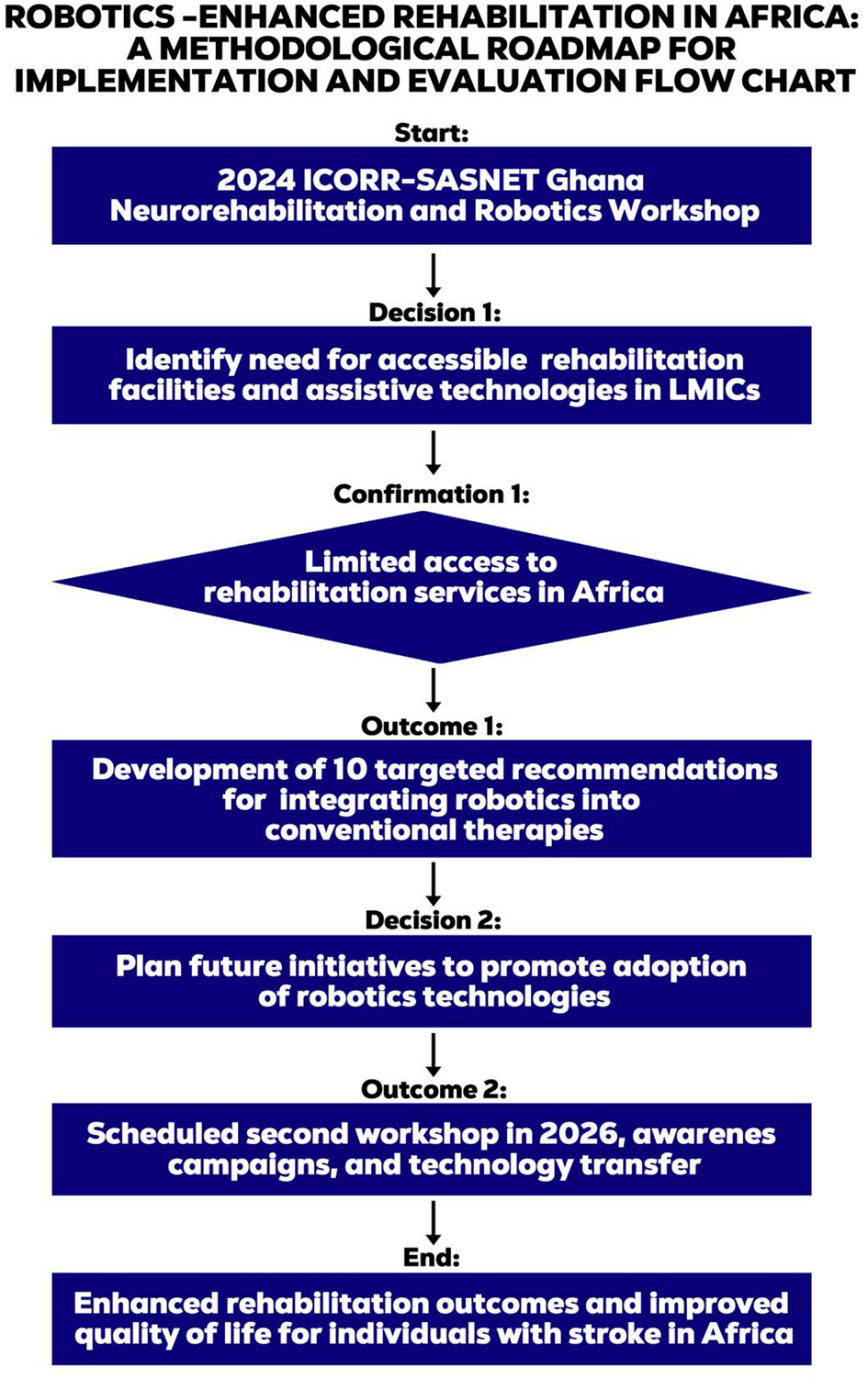
Flow diagram to illustrate the key decisions, confirmations, and outcomes from the event.

Key decisions included planning future initiatives to promote the adoption of robotics technologies, with outcomes including the scheduling of a second workshop in 2026, awareness campaigns, and technology transfer programs. Ultimately, this roadmap aims to enhance rehabilitation outcomes and improve the quality of life for individuals with stroke in Africa, leveraging robotics-enhanced rehabilitation to address the region's unique challenges. Table 3 lists these recommendations and Figure 4—An illustration of the roadmap of the outcome of the workshop.

## 5 Conclusions

The growing burden of stroke in LMICs demands immediate attention and action. The 2024 ICORR-SASNET-Ghana Neurorehabilitation and Robotics Workshop has taken a significant step toward addressing this challenge by developing ten targeted recommendations to integrate robotics technologies into mainstream conventional therapies in LMICs.

To translate these recommendations into tangible impact, SASNET-Ghana will collaborate with ICORR and key stakeholders to spearhead their implementation. Together, we can enhance rehabilitation outcomes and improve the lives of individuals living with stroke.

We call on policymakers, healthcare professionals, and technology experts to join forces in driving this initiative forward. Future directions include hosting a second workshop in 2026, launching awareness campaigns on robot-assisted rehabilitation, Assistive technology and facilitating technology transfer to African settings. By working together, we can make community based rehabilitation, neurorehabilitation, rehabilitation robotics and assistive technology more accessible and effective for those who need it most.

## Data Availability

The original contributions presented in the study are included in the article/Supplementary material, further inquiries can be directed to the corresponding authors.

## Footnotes

^1^https://tenvolunteers.org/ (accessed March 14, 2024).

^2^2021 Ghana Population and Housing Census Report on https://www.statsghana.gov.gh/.

^3^International Society of Wheelchair Professionals (ISWP). Available online at: https://iswp.org/.

^4^Technocare Innovation. Available online at: https://techcareinnovation.com.

## References

[B1] Ayalew A. T. Adane D. T. Obolla S. S. Ludago T. B. Sona B. D. Biewer G. (2020). From community-based rehabilitation (CBR) services to inclusive development. a study on practice, challenges, and future prospects of CBR in gedeo zone (Southern Ethiopia). Front. Educ. 5:506050. 10.3389/feduc.2020.506050

[B2] Basla C. Mariani G. Wolf P. Riener R. van Hedel H. J. (2024). Enhancing walking efficiency of adolescents with neurological impairments using an exosuit for ambulatory activities of daily living. Front. Robot. AI 11:1335733. 10.3389/frobt.2024.1335733 38549947 PMC10976852

[B3] Bouri M. Baur C. Clavel R. (2013). ““Handreha”: a new hand and wrist haptic device for hemiplegic children,” in ACHI 2013, The Sixth International Conference on Advances in Computer-Human Interactions, ed L. Miller (Nice: IARIA XPS Press), 286–292.

[B4] Bright T. Wallace S. Kuper H. (2018). A systematic review of access to rehabilitation for people with disabilities in low- and middle-income countries. Int. J. Environ. Res. Public Health 15:2165. 10.3390/ijerph15102165 30279358 PMC6210163

[B5] Calabrò R. S. Naro A. Russo M. Bramanti P. Carioti L. Balletta T.. (2018). Shaping neuroplasticity by using powered exoskeletons in patients with stroke: a randomized clinical trial. J. Neuroeng. Rehabil. 15, 1–16. 10.1186/s12984-018-0377-8 29695280 PMC5918557

[B6] Cyuzuzo C. Dukuzimana M. Muhire C. Sheldon Ames M. Ngwakongnwi E. (2025). Challenges to rehabilitation services in sub-Saharan Africa from a user, health system, and service provider perspective: scoping review. JMIR Hum. Fact. 12:e58841. 10.2196/58841 40019801 PMC11887585

[B7] Demofonti A. Carpino G. Zollo L. Johnson M. J. (2021). Affordable robotics for upper limb stroke rehabilitation in developing countries: a systematic review. IEEE Transact. Med. Robot. Bion. 3, 11–20. 10.1109/TMRB.2021.3054462

[B8] De-Rosende-Celeiro I. Torres G. Seoane-Bouzas M. (2019). Exploring the use of assistive products to promote functional independence in self-care activities in the bathroom. PLoS One 14:0215002. 10.1371/journal.pone.0215002 30958846 PMC6453482

[B9] Duncan P. W. Horner R. D. Reker D. M. Samsa G. P. Hoenig H. Hamilton B.. (2002). Adherence to postacute rehabilitation guidelines is associated with functional recovery in stroke. Stroke 33, 167–178. 10.1161/hs0102.101014 11779907

[B10] Ekechukwu E. N. D. Olowoyo P. Nwankwo K. O. Olaleye O. A. Ogbodo V. E. Hamzat T. K.. (2020). Pragmatic solutions for stroke recovery and improved quality of life in low-and middle-income countries—a systematic review. Front. Neurol. 11:337. 10.3389/fneur.2020.00337 32695058 PMC7336355

[B11] Gocevska M. Gjerakaroska-Savevska C. Nikolikj-Dimitrova E. Koevska V. Mitrevska B. Kalchovska B.. (2024). Robotic solutions for post-stroke recovery–literature review. Arch. Public Health 16, 67–70. 10.3889/aph.2024.6149

[B12] Haddadin S. (2014). Towards Safe Robots. Berlin; Heidelberg: Springer, 90. 10.1007/978-3-642-40308-8

[B13] Hartley S. Okune J. (2008). CBR Policy Development and Implementation. Norwich, University of East Anglia.

[B14] Husemann B. Muller F. Krewer C. Heller S. Koenig E. (2007). Effects of locomotion training with assistance of a robot-driven gait orthosis in hemiparetic patients after stroke: a randomized controlled pilot study. Stroke 38, 349–354. 10.1161/01.STR.0000254607.48765.cb 17204680

[B15] Johnson M. J. Keyvanian S. Mendonca R. J. (2024). “Toward inclusive rehabilitation robots,” in Rehabilitation Robots for Neurorehabilitation in High-, Low-, and Middle-Income Countries, eds M. J. Johnson and R. J. Mendonca (Academic Press), 471–498. 10.1016/B978-0-323-91931-9.00032-3

[B16] Johnson M. J. Mendonca R. (2022). Rehabilitation Robotics. Rehabilitation Engineering: Principles and Practice. Boca Raton, FL: CRC Press (Taylor and Francis Group). 10.1201/b21964-19

[B17] Johnson M. J. Mendonca R. J. (eds.). (2023). Rehaabilitation Robots for Neurorehabilitation in High-, Low-, and Middle-Income Countries: Current Prctice, Barriers, and Future Directions. Academic Press.

[B18] Johnson M. J. Rai R. Barathi S. Mendonca R. Bustamante-Valles K. (2017). Affordable stroke therapy in high-, low-and middle-income countries: From Theradrive to Rehab CARES, a compact robot gym. J. Rehabil. Assist. Technol. Eng. 4:2055668317708732. 10.1177/2055668317708732 31186929 PMC6453086

[B19] Kamban N. Norman R. (2012). Developing Low-Cost Assistive Technologies for Persons With Disabilities. ECHO Development Notes no. 117. North Carolina: ECHO International Health Services.

[B20] Kebaetse M. Johnson M. J. “Tsima B. Ocampo C. Nthitu J. M. Mogorosi N.. (2024). “Africa region: Botswana,” in Rehabilitation Robots for Neurorehabilitation in High-, Low-, and Middle-Income Countries, eds M. J. Johnson and R. J. Mendonca (Academic Press), 383–401. 10.1016/B978-0-323-91931-9.00018-9

[B21] Kimmatudawage S. P. Srivastava R. Kachroo K. Badhal S. Balivada S. (2024). “Toward global use of rehabilitation robots and future considerations,” in Rehabilitation Robots for Neurorehabilitation in High-, Low-, and Middle-Income Countries, eds M. J. Johnson and R. J. Mendonca (Academic Press), 499–516. 10.1016/B978-0-323-91931-9.00002-5

[B22] Komolafe M. A. Ayodele K. P. Olaogun M. O. Ogunbona P. O. Fawale M. B. Ogundele A. O.. (2024). Africa Region: Nigeria. Rehabilitation Robots for Neurorehabilitation in High-, Low-, and Middle-Income Countries, eds M. J. Johnson and R. J. Mendonca (Academic Press), 367–381. 10.1016/B978-0-323-91931-9.00008-6

[B23] Krebs H. I. Hamilton T. (2024). “Evidence for rehabilitation and socially assistive robotics,” in Rehabilitation Robots for Neurorehabilitation in High-, Low-, and Middle-Income Countries (Norwich: Academic Press), 67–94. 10.1016/B978-0-323-91931-9.00023-2

[B24] Lauretti C. Luzio F. S. D. Demofonti A. Tamantini C. Cordella F. Tagliamonte N. L. (2025). “Towards human-centric, sustainable, and resilient robot technologies,” in Healthcare in the Digital Age, eds. M. Bertolaso, M. L. Ilardo, and J. Ribera (Singapore: Palgrave Macmillan).

[B25] Li X. He Y. Wang D. Rezaei M. J. (2024). Stroke rehabilitation: from diagnosis to therapy. Front. Neurol. 15:1402729. 10.3389/fneur.2024.1402729 39193145 PMC11347453

[B26] Lunsford C. Grindle G. Salatin B. Dicianno B. E. (2017). Innovations with 3-dimensional printing in physical medicine and rehabilitation: a review of the literature. PM R. 8, 1201–1212. 10.1016/j.pmrj.2016.07.003 27424769

[B27] Manzoori A. R. Malatesta D. Primavesi J. Ijspeert A. Bouri M. (2024). Evaluation of controllers for augmentative hip exoskeletons and their effects on metabolic cost of walking: explicit versus implicit synchronization. Front. Bioeng. Biotechnol. 12:1324587. 10.3389/fbioe.2024.1324587 38532879 PMC10963600

[B28] Mitra S. Posarac A. Vick B. (2012). Disability and poverty in developing countries: a multidimensional study. World De. 41, 1–18. 10.1016/j.worlddev.2012.05.024

[B29] Naicker A. S. Htwe O. Tannor A. Y. De Groote W. Yuliawiratman B. S. Naicker M. S. (2019). Facilitators and barriers to the rehabilitation workforce capacity building in low- to middle-income countries. Phys. Med. Rehabil. Clin. N. Am. 30, 867–877. 10.1016/j.pmr.2019.07.009 31563176

[B30] Noukpo S. I. Kossi O. Triccas L. T. Adoukonou T. Feys P. (2022). Content and effectiveness of community-based rehabilitation on quality of life in people post stroke: a systematic review with meta-analysis. Disabil. CBR Incl. Dev. 33, 75–107. 10.47985/dcidj.57127885969

[B31] Olivier J. Jeanneret-Grosjean M. O. Bouri M. Bleuler H. (2014). “The LegoPress: A Rehabilitation, performance assessment and training device,” in Eurohaptics, eds M. Auvray and C. Duriez (Berlin: Springer). 10.1007/978-3-662-44196-1_25

[B32] Pollock A. Farmer S. E. Brady M. C. Langhorne P. Mead G. E. Mehrholz J.. (2014). Interventions for improving upper limb function after stroke. Cochr. Database Syst. Rev. 2014:CD010820 10.1002/14651858.CD010820.pub2 25387001 PMC6469541

[B33] Rodríguez-Fernández A. Lobo-Prat J. Font-Llagunes J. M. (2021). Systematic review on wearable lower-limb exoskeletons for gait training in neuromuscular impairments. J. Neuroeng. Rehabil. 18:22. 10.1186/s12984-021-00815-5 33526065 PMC7852187

[B34] Roy A. Mavuduri P. (2020). Chapter 22: future and impact of rehabilitation robotics on post-stroke care and recovery. Technol. Glob. Public Health 353–372. 10.1007/978-3-030-46355-7_27

[B35] Sahoo S. K. Choudhury B. B. (2023). A review on smart robotic wheelchairs with advancing mobility and independence for individuals with disabilities. J. Decis. Anal. Intell. Comp. 3, 221–242. 10.31181/10001122023s

[B36] Salvini P. Paez Granados D. Billard A. (2022). Safety concerns emerging from robots navigating in crowded pedestrian areas. Int. J. Soc. Robot. 14, 441–462. 10.1007/s12369-021-00796-4

[B37] Scorrano M. Ntsiea V. Maleka D. (2018). Enablers and barriers of adherence to home exercise programmes after stroke: caregiver perceptions. Int. J. Therapy Rehabil. 25, 353–364. 10.12968/ijtr.2018.25.7.353

[B38] Sukerkar K. Suratwala D. Saravade A. Patil J. D'britto R. (2018). Smart wheelchair: a literature review. Int. J. Inf. Commun. Technol. 7:63. 10.11591/ijict.v7i2.pp63-66

[B39] Tangcharoensathien V. Witthayapipopsakul W. Viriyathorn S. Patcharanarumol W. (2018). Improving access to assistive technologies. WHO South East Asia J. Public Health 7, 84–89. 10.4103/2224-3151.239419 30136666

[B40] Tannor A. Y. Nyarko F. K. A. Quao B. O. Adams E. A. (2024). “Africa region: Ghana,” in Rehabilitation Robots for Neurorehabilitation in High-, Low-, and Middle-Income Countries, eds M. J. Johnson and R. J. Mendonca (Academic Press), 403–418. 10.1016/B978-0-323-91931-9.00003-7

[B41] Wagner T. H. Lo A. C. Peduzzi P. Bravata D. M. Huang G. D. Krebs H. I.. (2011). An economic analysis of robot-assisted therapy for long-term upper-limb impairment after stroke. Stroke 42, 2630–2632. 10.1161/STROKEAHA.110.606442 21757677 PMC4445835

[B42] World Health Organization (1978). Primary health care: Report of the International Conference on Primary Health Care, Alma-Ata, USSR, 6-12 September 1978. Geneva: World Health Organization. Available online at: https://apps.who.int/iris/handle/10665/39228

[B43] World Health Organization (2010). Community-Based Rehabilitation: CBR Guidelines. World Health Organization. Available online at: https://www.who.int/publications/i/item/9789241548052 (Accessed July 23, 2024). 26290927

[B44] World Health Organization (2024). Intersectoral Global Action Plan on Epilepsy and Other Neurological Disorders 2022–2031: Implementation Toolkit. Geneva: World Health Organization. Licence: CC BY-NC-SA 3.0 IGO. Available online at: https://www.who.int/publications/i/item/9789240076624 (Accessed July 23, 2024).

[B45] Yang G. Z. Riener R. Dario P. (2017). To integrate and to empower: robots for rehabilitation and assistance. Sci. Robot. 2:eaan5593. 10.1126/scirobotics.aan5593 33157873

[B46] Zuurmong M. O'Banion D. Gladstone M. Carsamar S. Kerac M. Baltussen M.. (2018). Evaluating the impact of a community based parent training programme for children with cerebral palsy in Ghana. PLoS ONE. 13:e0202096. 10.1371/journal.pone.0202096 30180171 PMC6122808

