# Supplementary material for: Pragmatic recommendations to improve access to rehabilitation robots, assistive technologies and neurorehabilitation services in Africa: proceedings from ICORR-SASNET Ghana neurorehabilitation workshop, 2024
Source: Front Stroke. 2025 Sep 1;4:1565651. doi: 10.3389/fstro.2025.1565651 (PMC12802663; doi:10.3389/fstro.2025.1565651)
Supplement: Supplementary file 1 [file Supplementary_file_1.pdf]

1. **Figure of Attendees**

Numbers	Institution/Organization	Designation
1.	Tikkun Olam Empowerment Network (TEN)-Winneba Rehab Project, Ghana	Physiotherapy Technician (PTT)
2.	Stroke Association Supportnetwork-Ghana (SASNET GHANA)	Community-based Rehabilitation Assistant and Social worker
3.	Tikkun Olam Empowerment Network (TEN)-Winneba Rehab Project, Ghana	CBR Professional & Physiotherapy Assistant
4.	Ghana Non Communicable Disease Alliance	Research Officer
5.	Korle Bu Teaching Hospital, Accra -Ghana	Stroke Nurse
6.	Michael and Francisca Foundation, Nigeria	Patients Lead and Advocate
7.	Ministry of Health, Ghana	Physiotherapist
8.	Stroke Association Supportnetwork-Ghana (SASNET GHANA) /Ghana Health Service	Community-based rehabilitation project Assistant/Health administrator
9.	Sogakope Hospital , Ghana	Physiotherapist
10.	Ghana Health Service	Physiotherapist
11.	Tikkun Olam Empowerment Network (TEN)-Winneba Rehab Project, Ghana	Physiotherapist
12.	Tikkun Olam Empowerment Network (TEN)-Winneba Rehab Project , Ghana	Physiotherapy Assistance
13.	Tikkun Olam Empowerment Network (TEN)-Winneba Rehab Project, Ghana	Physiotherapist

14.	Tikkun Olam Empowerment Network (TEN)-Winneba Rehab Project , Ghana	Occupational-Therapy Assistant
15.	Tikkun Olam Empowerment Network (TEN)-Winneba Project, Ghana	Director TEN Rehab Project
16.	Heart Hands and Voice (HHVF) Foundation	Community –Based Rehabilitation Practitioner
17.	Organization of Social Intervention in Development (OSID)-ACCRA	Social worker
18.	University of Medical Sciences, Ondo Nigeria	Lecturer/ Medical Rehabilitation Professional
19.	Heart Hands and Voice (HHVF) Foundation	Caregiver
20.	Heart Hands and Voice (HHVF) Foundation	Manager
21.	Tikkun Olam Empowerment Network (TEN)-Winneba Project , Ghana	Occupational Therapist
22.	Tikkun Olam Empowerment Network (TEN)-Winneba Rehab Project , Ghana	Physiotherapist
23.	Tikkun Olam Empowerment Network (TEN)-Winneba Rehab Project , Ghana	Occupational Therapist
24.	Ghana Health Service	Physiotherapist
25.	Ghana Non Communicable Disease Alliance	Communication Officer
26.	Tikkun Olam Empowerment Network (TEN)-Winneba Rehab Project, Ghana	Physiotherapist
27.	Church of Pentecost Rehabilitation Center	Manager
