# Supplementary material for: Pragmatic recommendations to improve access to rehabilitation robots, assistive technologies and neurorehabilitation services in Africa: proceedings from ICORR-SASNET Ghana neurorehabilitation workshop, 2024
Source: Front Stroke. 2025 Sep 1;4:1565651. doi: 10.3389/fstro.2025.1565651 (PMC12802663; doi:10.3389/fstro.2025.1565651)
Supplement: Supplementary file 2 [file Supplementary_file_2.pdf]

The Thematic Areas of the Presentations and Speakers:

1. **Status of stroke rehabilitation in Africa and Ghana and Policies driving change**

   Emmanuel Aggrey Nelson, Shani Halfon, Mary A. Agoriwo, Sserunkuma C. Maholo, Prof. Mayowa O. Owolabi

2. **The role of Assistive Technology and Rehabilitation Devices in Africa and Ghana**

   Dr. Leslie Ajavon, Dr. Benedict Okoe Quao, Mr. Alex Kamadu

3. **Rehabilitation Robotic Systems and potential strategies to increase inclusivity**

   Prof. Dr. Dr. h.c Robert Reiner, Prof. Michelle J. Johnson, Prof. Isabel Gunther ,Mary Collier Barnes, Dr. Khor Kang Xiang, Prof Mohamad Bouri, Prof Matthew O. Olaogun, Prof. Morenikeji A. Komolafe, Chinonso Amanda Ad Adams, Prof. Kayode Ayodele, Dr. Ahmad A. Sanusi, Dr. Ahmed Omokayode Idowu , Dr. Adebimpe Ogunmodede, Dr. Uchenna Chidi Eke, Ayenowowon Sunday Olaoluwa
