# Supplementary material for: Pragmatic recommendations to improve access to rehabilitation robots, assistive technologies and neurorehabilitation services in Africa: proceedings from ICORR-SASNET Ghana neurorehabilitation workshop, 2024
Source: Front Stroke. 2025 Sep 1;4:1565651. doi: 10.3389/fstro.2025.1565651 (PMC12802663; doi:10.3389/fstro.2025.1565651)
Supplement: Supplementary file 3 [file Supplementary_file_3.pdf]

ICORR | MINISTRY OF HEALTH REPUBLIC OF GHANA | GHANA HEALTH SERVICE — Your Health – Our Concern | NEUROLOGY SOCIETY OF GHANA | ETH4D | SASNET GHANA

THE STROKE ASSOCIATION SUPPORTNETWORK - GHANA (SASNET - GHANA), INTERNATIONAL CONSORTIUM FOR REHABILITATION ROBOTICS (ICORR)

In partnership with

MINISTRY OF HEALTH (MOH), GHANA HEALTH SERVICE (GHS)
NEUROLOGY SOCIETY OF GHANA (NSG)
ETH FOR DEVELOPMENT (ETH 4D)

presents

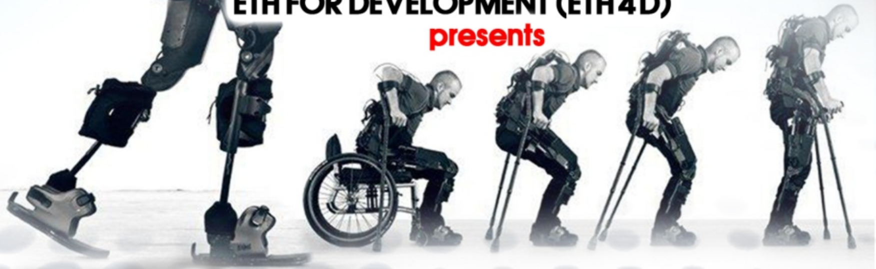

2 DAYS ICORR NEUROREHAB AND ROBOTICS TRAINING WORKSHOP

Title: Community - Based Neurorehabilitation & Robotics In Lower - Middle Income Countries (LMIC's)

SPEAKERS

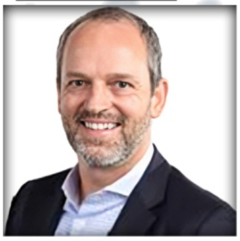

PROF. ROBERT RIENER

Professor of Rehab Robotics and Medicine at ETH and University Zurich, President of ICORR

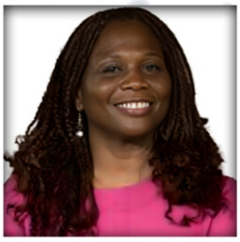

PROF. MICHELLE J. JOHNSON

Associate Professor in Bioengineering and in Mechanical Engineering and Applied Mechanics, University of Pennsylvania, USA

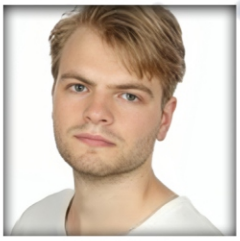

DOMINIK WOJCIKIEWICZ

Sensory-Motor Systems Laboratory , ETH Zürich, Switzerland

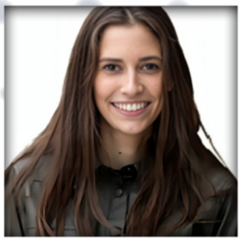

CHIARA BASLA

Sensory-Motor Systems Laboratory, ETH Zürich, Switzerland

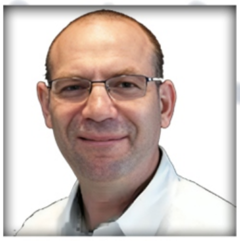

PROF. MOHAMED BOURI

Head, research group of Rehabilitation and Assistive Robotics (REHAssist) at the Ecole Polytechnique Fédérale de Lausanne (EPFL)

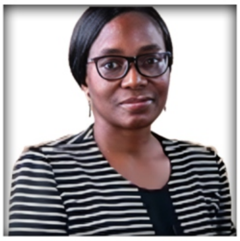

PROF MORENIKEJI KOMOLAFE

Professor of Neurology at the Obafemi Awolowo University, Ile-Ife, Nigeria

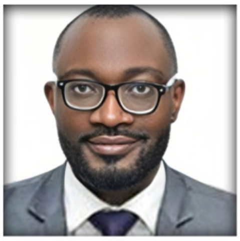

PROF. MAYOWA OWOLABI

Professor of Neurology; Pioneer Director, Center for Genomic and Precision Medicine, College of Medicine, University of Ibadan Foundation co-chair of the steering committee of African Stroke Organization (ASO)

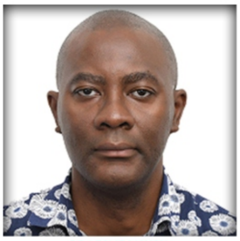

DR. LESLIE AJAVON

African fellow of the International Rehabilitation Forum (IRF) Member, World Rehabilitation Alliance(WHO) Primary Care Work-stream group advocating for the integration of Rehabilitation services into primary care.

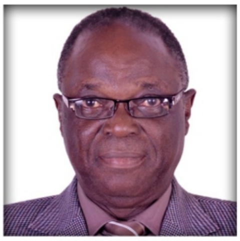

PROFESSOR MATTHEW OLATOKUNBO BAMIDELE OLAOGUN,

BSc (Ib), MS (S.Fraser), FNPC (Neuro & Mental Health), MNIM Professor of Physiotherapy, University of Medical Sciences, Ondo, Nigeria.

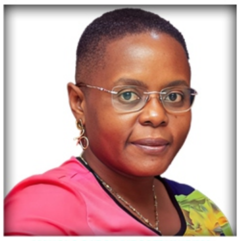

MAHOLO CAROLYNE SSERUNKUMA

Treasurer, Community Based Rehabilitation (CBR) Africa Network

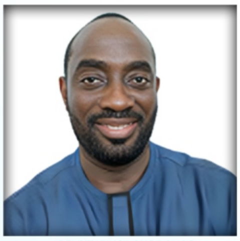

DR. BENEDICT OKOE QUAO

Sport, Exercise and Rehab fellow, Family Medicine Specialist and Global Health practitioner

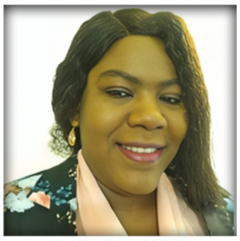

MARY WETANI AGORIWO

SPT. MSc. PhD (Candidate) Neuro-physiotherapist, Assistant Lecturer at the University of Health and Allied Sciences, Ho, Ghana

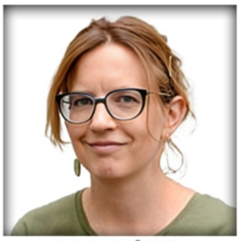

PROF. ISABEL GÜNTHER

Professor of Development Economics and Academic Director of NADEL ETH Zürichnd, Switzerland

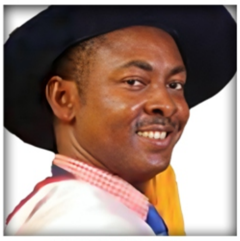

DR. KAYODE P. AYODELE

Ph.D. in Instrumentation and Control Engineering, Obafemi Awolowo University, Nigeria

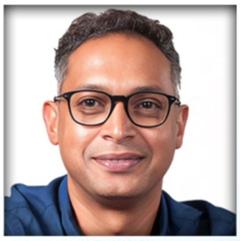

ALEX KAMADU

Executive Director of the International Society of Wheelchair Professionals (ISWP)

Expert in fields of occupational therapy, health system strengthening and postural seating

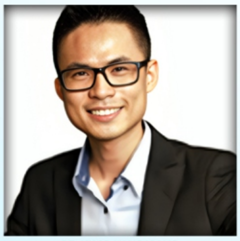

DR. KHOR KANG XIANG

Founder, and Rehab Technology Specialist at Techcare Innovation

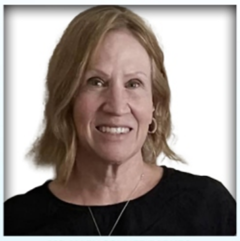

MS. MARY COLLIER BARNES

MOT, CHT, CIDN

15TH-16TH MARCH, 2024

@DR. JOSEPH OKIE GOGO HALL CSIR - STEPRI NEAR ALLIANCE FRANCIASE, ACCRA- GHANA

2 DAYS ICORR NEUROREHAB AND ROBOTICS TRAINING WORKSHOP

Title: Community - Based Neurorehabilitation & Robotics In Lower-Middle Income Countries (LMIC's)

WELCOME NOTE

On behalf of the planning team and in my capacity as the Executive director of Stroke Association Supportnetwork - Ghana (SASNET GHANA) and the coordinator for ICORR Neurorehab and Robotics workshop 2024, I welcome you all to the three-days training workshop themed, 'Neurorehabilitation and Robotics in Lower-Middle-Income Countries (LMICs)'. This training workshop which takes place in the capital city of Ghana, is organized by the Stroke Association Supportnetwork – Ghana (SASNET-GHANA) and the International Consortium for Rehabilitation Robotics (ICORR) in partnership with the Ministry of Health (MOH) Ghana, Ghana Health Service and the Neurology Society of Ghana (NSG).

Ghana is bounded on the north by Burkina Faso, on the east by Togo, on the south by the Atlantic Ocean, and on the west by Côte D'Ivoire. Formerly a British colony known as the Gold Coast was led to independence by Dr. Kwame Nkrumah on the 6th of March, 1957. Ghana became the first black nation in sub-Saharan Africa to achieve independence from colonial rule and a gateway to Africa and ranked as a peaceful country in Africa. The country is named after the ancient empire of Ghana, from which the ancestors of the inhabitants of the present country are thought to have migrated

The workshop is packed with latest topics by International experts in neurorehabilitation and robotics. There will be interactive sessions for discussions and practical demonstrations.

Accra, the capital city of Ghana, is accommodating and very peaceful to live in with a lot of tourist sites such as the, Dr. Kwame Nkrumah Mausoleum (Late President of Ghana), Osu Castle, Independence Square, Aburi Gardens, Labadi Beach, etc. Ga is the language for the indigenous people of Accra. Please enjoy your stay, places of interest while you are in Accra.

Akwaaba

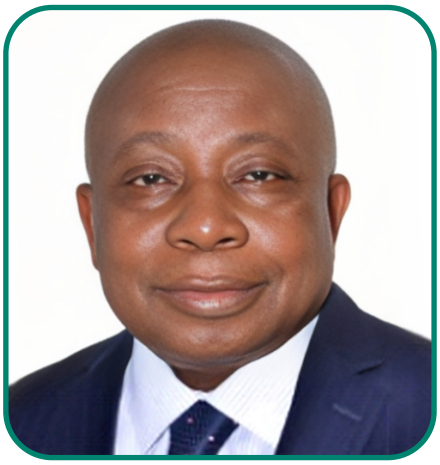

Outgoing Minister of Health,
Hon. Kwaku Agyeman-Manu

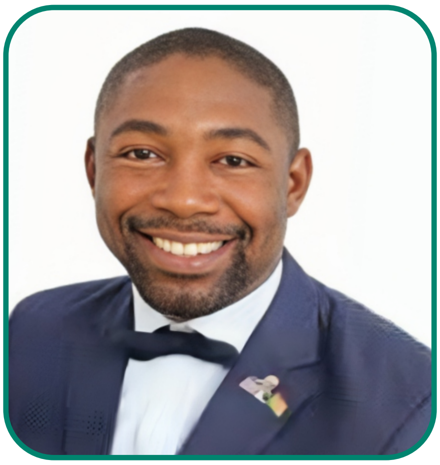

Health Minister - Designate
Hon. Dr Bernard Okoe Boye

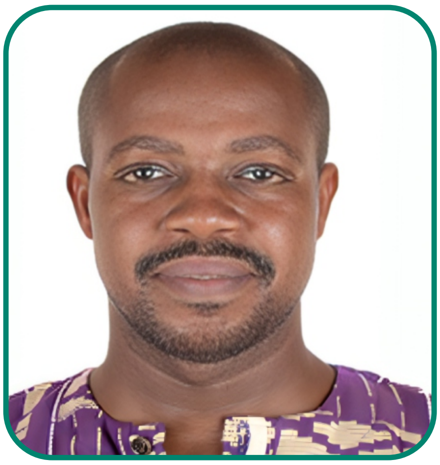

Ad Adams Ebenezer,
Executive Director
SASNET GHANA,
Coordinator, Neurorehab &
Robotics Training Workshop 2024

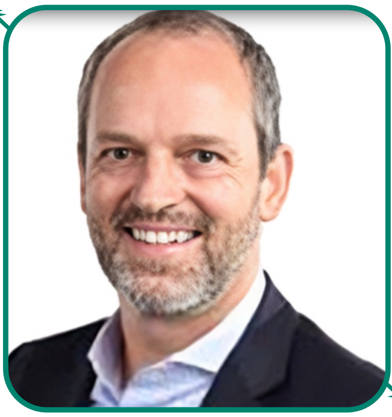

PROF. ROBERT RIENER

Professor of medicine at the medical faculty of the University of Zurich.
President, International Consortium of Rehabilitation Robotics (ICORR).

Robert Riener studied Mechanical Engineering at TU München, Germany, and University of Maryland, USA. He received a Dr.-Ing. degree in Engineering from the TU München in 1997. After postdoctoral work from 1998-1999 at the Centro di Bioingegneria, Politecnico di Milano, he returned to TU München, where he completed his Habilitation in the field of Biomechatronics in 2003. In 2003 he became assistant professor at ETH Zurich and Spinal Cord Injury Center of the University Hospital Balgrist ("double-professorship"); since 2010 he has been full professor for Sensory-Motor Systems at ETH Zurich, and since 2016 also full professor of medicine at the medical faculty of the University of Zurich. Since 2012, Riener is member of the Department of Health Sciences and Technology, which he was chairing as head 2016 – 2018. He was guest professor at USC Los Angeles and SSSA Pisa, and he still is at SJTU Shanghai. Riener has published more than 500 peer-reviewed journal and conference articles, 36 books and book chapters and filed 26 patents. He has received 26 personal distinctions and awards including the Swiss Technology Award in 2006, the IEEE TNSRE Best Paper Award 2010, and the euRobotics Technology Transfer Awards 2011, 2012, and 2021.

Riener's research focuses on the investigation of the sensory-motor interactions between humans and machines. This includes the development of powered prostheses, exoskeletons, and wheelchairs as well as user-cooperative robotic devices and virtual reality technologies applied to neurorehabilitation. Riener is the initiator and organizer of the CYBATHLON, which was honored with the European Excellence Award, the Yahoo Sports Technology Award and two REIMAGINE Education Awards. In 2018 Riener obtained the honorary doctoral degree from the University of Basel. In 2022 Riener became president of the ICORR (International Consortium of Rehabilitation Robotics). Since 2019, Riener is AAAS Leshner Leadership Fellow and since 2023 he is member of the Swiss Academy of Technical Sciences (SATW).

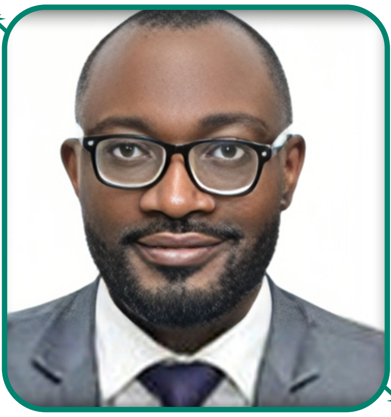

PROF. MAYOWA OWOLABI

Professor of Neurology; pioneer Director, Center for Genomic and Precision Medicine, College of Medicine, University of Ibadan

Mayowa OWOLABI FMCP, FWACP, FAAN, FANA, FAHA, FRCP, FAAS, FAMedS, FAS Professor of Neurology; pioneer Director, Center for Genomic and Precision Medicine, College of Medicine, University of Ibadan; Honorary Consultant Neurologist, University College Hospital, Ibadan, Nigeria, is an eminent scholar with a stroke phenotyping software patent; >400 publications in peer-reviewed journals including The Lancet, and Nature, with >126,000 citations and a Google Scholar h-index of 85. Prof. Owolabi is a recognized global leader in medicine, neurology, cardiovascular diseases, neuro-rehabilitation, global health, brain health, community-based genomic epidemiology of stroke in Africa, clinical trials and implementation science. He is an outstanding scientist with several inventions and innovations including the 'Seed of Life Model' a conceptual model of holistic essence and quality of life; the HRQOLISP, a multiculturally-validated quality of life measure for stroke, available globally in several versions and languages; 'stroke quadrangle' and 'brain quadrangle', which are accepted globally as key ingredients driving the global interventions against stroke and for promoting brain health in Africa and beyond; 'stroke levity scale' , a simple brief and valid stroke severity scale; 'stroke recovery spiral' a pathway to recovery after stroke tapping internal adaptation and psycho-spiritual wellbeing; 'cervical vertigo tetrad'; and implementation cycle for translating evidence into policy and practice in the control of chronic diseases. He unraveled the dominant risk genetic and non-genetic risk factors for stroke in Africa and discovered the protective effect of green leafy vegetables against stroke and hypertension. He led the SIREN team as the first to discover the association between APOL 1 and small vessel disease stroke. He also led the discovery of genome-wide association of microRNA and stroke in Africans. He led the development of prediction models for stroke and hypertension in Africa.

He is among the global top 2% scientists (2023) and the winner of the 2021 World Stroke Organization Global Award for Outstanding Contributions to Clinical Stroke Research. He is a foremost leader in the global fight against stroke and a frontline leader in the fight against hypertension, stroke and non-communicable diseases in Africa. He is currently leading the implementation call for action against hypertension in Africa working with the World Hypertension League, World Health Organization and Resolve To Save Lives. He is associate editor of Stroke journal and several other top tier Neurology journals. He leads the largest stroke study in Africa as principal investigator of several grants and co-investigator in several (with >$80 million overall) including Stroke Investigative Research & Educational Network (SIREN) grant (with the largest neurobiobank in Africa > 170,000 samples) from National Institutes of Health (NIH), USA; Systematic Investigation of Blacks with Stroke using Genomics (SIBS Genomics) R01NS107900. He is the pioneer Chair of the largest study of cardiovascular diseases in Africa (H3Africa-CVD WG with >55,000 subjects). He is the pioneer Regional Vice-President, World Federation of Neurorehabilitation (Africa); member Board of Directors, World Stroke Organization; member African Regional Director, World Hypertension League; Lead Co-Chair, WSO- Lancet Commission on stroke which just published the pragmatic solutions to reduce the burden of stroke globally. He established the accelerating African Control of Hypertension through Innovative Epidemiology and a Vibrant Ecosystem (ACHIEVE) and is Co-Lead Africa-Europe CoRE in Non-Communicable Diseases & Multimorbidity, African Research Universities Alliance ARUA & The Guild. He is foundation co-chair of the steering committee of African Stroke Organization; and Rapporteur/Member of the WHO Technical Action Group on NCD (Research and Innovation).

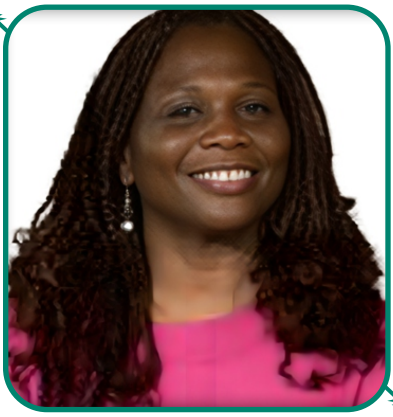

PROF. MICHELLE J. JOHNSON, PHD

Associate professor in Bioengineering and in Mechanical Engineering and Applied Mechanics, University of Pennsylvania, USA.

Michelle J. Johnson, Ph.D., is currently Associate professor of Physical Medicine and Rehabilitation at the University of Pennsylvania. She has secondary appointments as an Associate professor in Bioengineering and in Mechanical Engineering and Applied Mechanics. She has a Bachelor of Science in Mechanical Engineering and Applied Mechanics from the University of Pennsylvania and a PhD in Mechanical Engineering, with an emphasis in mechatronics, robotics, and design, from Stanford University. She completed a NSF-NATO post-doctoral fellowship at the Advanced Robotics Technology and Systems Laboratory at the Scuola Superiore Sant'Anna in Italy. She directs the Rehabilitation Robotic Research and Design Laboratory located at the Pennsylvania Institute of Rehabilitation Medicine at the University of Pennsylvania, School of Medicine. The lab is also affiliated with the General Robotics Automation Sensing Perception (GRASP) Lab. Dr. Johnson's lab specializes in the design, development, and therapeutic use of novel, affordable, intelligent robotic assistants for rehabilitation in high and low-resource environments with an emphasis on using robotics and sensors to quantify upper limb motor function in adults and children with brain injury or at risk for brain injury. Dr. Johnson has spent over twenty years applying technology solutions to aid in the understanding of disability and impairment after brain injury. She is currently a Fulbright Scholar for 2020-2022 to Botswana and an IEEE Engineering in Biology and Medicine Society Distinguished Lecturer 2021-2022.

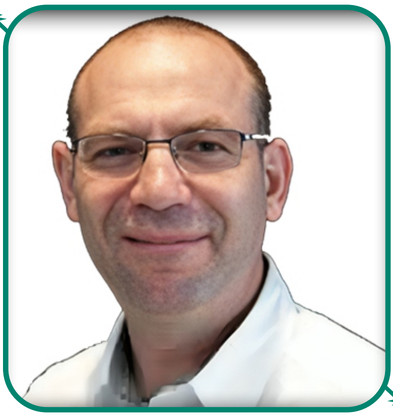

PROF. MOHAMED BOURI

Head, research group of Rehabilitation and Assistive Robotics (REHAssist) at the Ecole Polytechnique Fédérale de Lausanne (EPFL).

Dr. Mohamed Bouri graduated from Ecole Nationale Polytechnique of Algiers and obtained his PhD thesis in 1997 from INSA-Lyon (Fr). He currently heads the research group of Rehabilitation and Assistive Robotics (REHAssist) at the Ecole Polytechnique Fédérale de Lausanne (EPFL). Mohamed Bouri is a roboticist specializing in developing and controlling complex robotics structures. His expertise in advanced industrial and medical robotics is established and recognized. He focuses on "Medical robotics" for rehabilitation, lower limb exoskeletons, and surgical applications. He is a board member of the International Consortium of Rehabilitation Robotics (ICORR) and on the board of directors of the Swiss Cobotics Center (S3C).

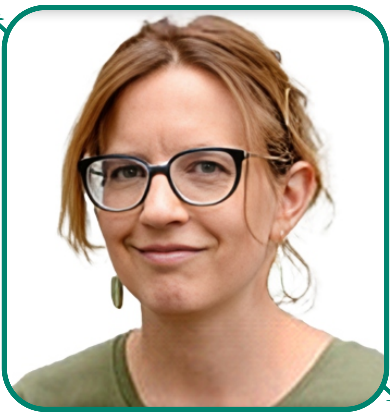

PROF. ISABEL GÜNTHER

Professor of Development Economics and Academic Director of NADEL ETH Zürichnd, Switzerland

Isabel Günther has been Professor of Development Economics and academic director of NADEL since July 2014. She had previously been Assistant Professor for Development Economics at ETH Zürich, research associate at the Faculty of Economics of the University of Göttingen and at the Harvard School of Public Health. Her main research interest is in empirical microeconomics with a particular focus on measurement of poverty and inequality, population economics, technologies for poverty reduction, and evidence-based policy making. Isabel Günther has carried out most of her research in Benin, Burkina Faso, Ghana, Kenya, Uganda, and South Africa."

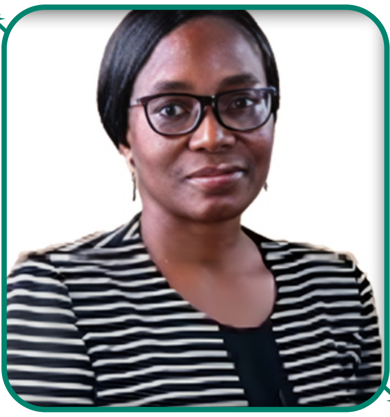

PROF. MORENIKEJI KOMOLAFE

Professor of Neurology at the Obafemi Awolowo University, Ile-Ife, Nigeria

Professor of Neurology at the Obafemi Awolowo University, Ile-Ife, Nigeria, Head Department of Medicine. Principal investigator on TETFUNFD NRF on Robotic rehabilitation in stroke. She has 80 published research papers to her credit. She received her Medical Degree at the University of Ibadan in 1990, Fellowship of the West African College of Physicians in Neurology in April 2000 and Diploma in Clinical Neurology at the Institute of Neurology, Queen Square London in 2009. She is the recipient of numerous awards such as: Third Place in the Stroke Day 2010. Awards recognition initiative, World Stroke Organization, World Sleep Day Distinguished activity Award 2022 and 2023.

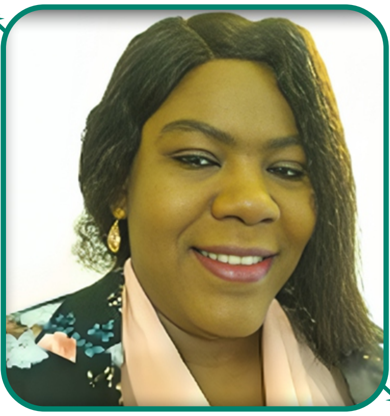

MARY WETANI AGORIWO

PT. MSc. PhD (Candidate)
Neuro-physiotherapist, assistant lecturer at the University of Health and Allied Sciences, Ho, Ghana

Mary is a neuro-physiotherapist with over a decade experience in clinical practice at the 37 Military and Korle Bu Teaching hospitals. She holds a BSc. in Physiotherapy from the University of Ghana and an MSc. in Neurorehabilitation from Brunel University London, UK. Mary is an assistant lecturer at the University of Health and Allied Sciences, Ho, Ghana and currently pursuing a PhD in Physiotherapy at the Stellenbosch University, South Africa. She is a member of the Ghana Physiotherapy Association (GPA) Evidence-Based Practice Group. She serves as a Co-Chair of the MDS-Africa Section Education Committee, coordinator of the Parkinson's Disease (PD) Support Group Ghana and served as a Co-director for the MDS-Africa Section Train the Trainer Pilot Program in 2022-2023. Mary is a former Chevening Scholar (2016/2017 Cohort), a 2021 Fellow of the Organization for Women in Science for the Developing World (OWSD) and 2023 Mawazo Fellow. Her research interest lies in identifying factors that influence the quality of care, especially rehabilitation, for persons with neurological conditions in Ghana and developing strategies to mitigate rehabilitation related problems.

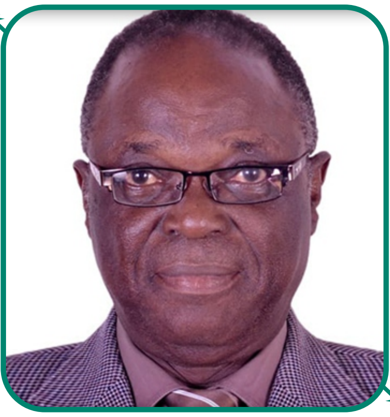

PROFESSOR MATTHEW OLATOKUNBO BAMIDELE OLAOGUN

BSc(Ib), MS(S.Fraser), FNPC(Neuro & Mental Health), MNIM, Professor of Physiotherapy, University of Medical Sciences, Ondo, Nigeria.

Professor Olaogun is a consultant physiotherapist/pathokinesiologist and specializes in Neurophysiotherapy and Neurorehabilitation including Robotic Neurorehabilitation.

He was a former Dean of Faculty of Basic Medical Sciences, Obafemi Awolowo University, IleIfe, Nigeria. He retired from the University in May, 2018. He was a visiting Scholar to the School of Allied Health Sciences, College of Health Sciences, University of Ghana, Korle-Bu, (July 2008- January, 2010). He was pioneer Dean of Faculty of Basic Medical and Health Sciences, Bowen University, Iwo. He also served as Senior Assistant Provost of the College of Health Sciences of the University. He was appointed Professor of Physiotherapy in the University of Medical Sciences, Ondo, August 25, 2021 and became the pioneer Dean of Faculty of Medical Rehabilitation of the University on assumption of duty.

Professor Olaogun was a co-investigator in the US NIH funded project titled 'Capacity building in sub-Saharan to conduct a cutting-edge genetics research in Parkinson's disease including the recruitment of Nigerian and South African patients'. He is currently a co-investigator in TETFUND NRF Research Project on a Robotic Platform for Upper Limb Stroke Rehabilitation for Improved Post-Stroke Functional Outcomes at Obafemi Awolowo University, Ile Ife.

He is the current President of Nigeria Federation of Neurorehabilitation.

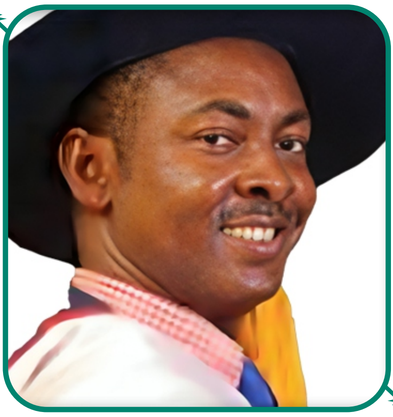

DR. KAYODE P. AYODELE

Ph.D. in Instrumentation and Control Engineering,
Obafemi Awolowo University, Nigeria

Dr. Kayode P. Ayodele is an Associate Professor of Electronic and Electrical Engineering, holding a Ph.D. in Instrumentation and Control Engineering from Obafemi Awolowo University, Nigeria. He currently serves as the Co-Director of the Applied Artificial Intelligence and Robotics Research Lab within the same university's Department of Electronic and Electrical Engineering.

His research over the last twenty-five years has focused on linear and nonlinear analysis and control of distributed instrumentation and robotic systems, with a very strong slant towards biomedical applications and, more recently, artificial intelligence models and techniques. Since 2018, he has been involved in the development of context-appropriate rehabilitation robots for developing countries. These efforts aim to advance the field of physical therapy and provide frugal, pragmatic, and effective solutions for individuals with mobility challenges in resource-constrained areas.

Dr. Ayodele's work has earned him recognitions such as a Carnegie iLab Junior Research Fellowship, the MIT-Total Empowering the Teachers Fellowship, and the IEEE Education Society Theodore L. Batchman Outstanding Paper Award.

As an educator, Dr. Ayodele is deeply committed to fostering the next generation of engineers and researchers. He has been involved in numerous teaching initiatives, focusing on enhancing engineering education in Nigeria. He played a pivotal role in the OAU iLabs remote laboratory architecture research group which pioneered the development of remote laboratories in Africa. His teaching philosophy emphasizes the practical application of theoretical knowledge, preparing students to tackle real-world challenges. An active member of several professional organizations, including the IEEE, the Society for Neuroscience, and the International Consortium of Rehabilitation Robotics, Dr. Ayodele continues to contribute significantly to his field.

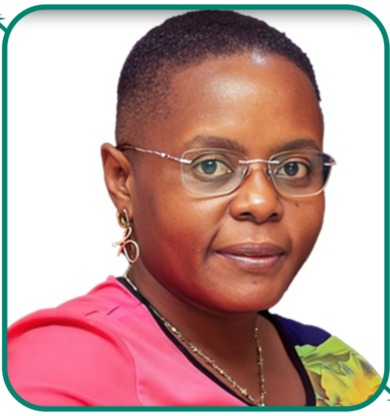

MS. MAHOLO CAROLYNE SSERUNKUMA

Treasurer CBR Africa Network and Executive Member CBR Global Network

Expert in Disability, Rehabilitation, Resilience Building and Inclusive Development

20yrs of experience, lecturing in the Department of Community and Disability Studies, Kyambogo University

Developed Disability Blueprint for Uganda, Zero Leprosy Roadmap Ethiopia and participated in developing the Zero Leprosy Roadmap for Uganda and the Zero Leprosy Roadmap for and Buruli Ulcer, Nigeria. Candidate PhD Disability Studies and Rural Development

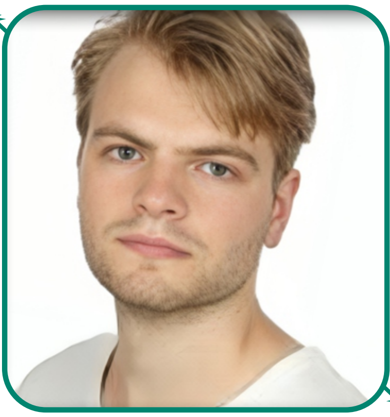

MR. DOMINIK WOJCIKIEWICZ

Sensory-Motor Systems Laboratory, ETH Zürich, Switzerland. In 2019, Dominik graduated from Jagiellonian University with a bachelor's degree in interdisciplinary sciences. Then, in 2023, he graduated from ETH Zurich with a Master's degree in Micro- and Nanosystems. During his studies, he has been conducting research on the intersection of physics, sensing, and computer science.

Alongside his academic pursuits, Dominik has gained experience through internships and research projects. At the Paul Scherrer Institute, he contributed to the development of a state-of-the-art grating-interferometry breast diagnosis system. During his Master's degree, he worked as a research assistant at ETH, where he developed software for a DIY spray coater using 3D printing technology. Additionally, he interned at Sensirion, working on an Internet of Things (IoT) project focused on methane leak monitoring. Dominik completed his Master's thesis in collaboration between the Robotic Systems Lab (RSL) and the Micro- and Nanosystems group (MNS). The thesis aimed to apply capacitive fingers, developed by MNS, to robotic grippers used in RSL. In May 2023, Dominik began his doctoral studies at the SCAI Lab. His research focuses on autonomous wheelchair navigation, encompassing hardware integration, machine learning-based user intention detection for shared navigation, and machine vision for obstacle avoidance.

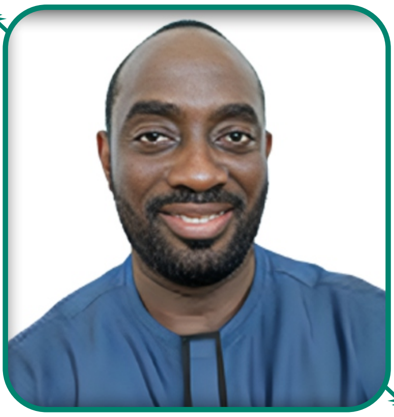

DR. BENEDICT QUAO

Sport, Exercise and Rehab fellow, Family Medicine Specialist and Global Health practitioner Family Medicine Specialist and Global Health practitioner with expertise in the programmatic control of leprosy and other skin-related neglected tropical diseases, and rehabilitation care, with more than a decade's post-qualification experience in a variety of clinical and healthcare managerial settings. As the national leprosy programme manager, he's been instrumental in Ghana's progressive integrated skin-NTD approach implementation, bringing on board a deep understanding of the workings at the public health-primary care interface and across the clinical-managerial spectrum. He also serves on the Leadership Team of the Global Partnership for Zero Leprosy, representing country leprosy programmes/Ministries of Health

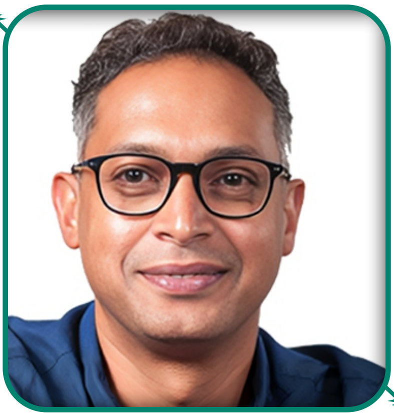

MR. ALEXANDER KAMADU

Executive Director of the International Society of Wheelchair Professionals (ISWP)

Alexander Kamadu has 23 years of combined international experience in the fields of occupational therapy, health system strengthening and postural seating. For many years he worked in the United Kingdom in the rehabilitation sector as an Occupational Therapist across acute and rehab settings and occupied strategic roles in the Department of Health and NHS England. Alex stands up for any social injustices and has a particular interest in improving the access and quality of wheelchair and assistive technology services globally. He is currently the Executive Director of the International Society of Wheelchair Professionals (ISWP) which is a society based in the USA that aims to coordinate all global wheelchair improvement efforts and promotes training and advocacy for all wheelchair providers. Prior to joining ISWP, he was involved in health system strengthening across sub Saharan Africa and has led research projects in the fields of HIV, social protection, sexual reproductive health, gender-based violence and LGBTQI+ issues. He is adept at connecting and communicating whilst engaging strategic, multi-sectoral partnerships with public and private entities including governments, bilateral & multilateral development agencies, donor organizations and civil society.

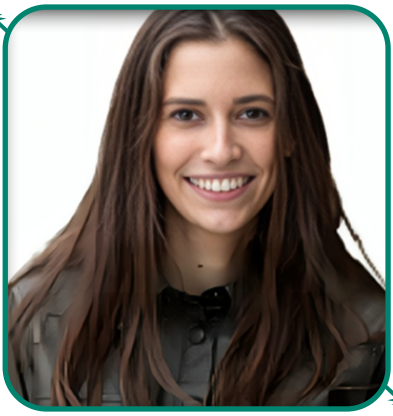

MS. CHIARA BASLA

Chiara received her Bachelor's and Master's degree in Biomedical Engineering at the Politecnico di Milano (Italy), with a specialization in Technologies for Electronics. She graduated in December 2020. She carried out her Master thesis at the Neuroengineering Laboratory (ETH Zürich, Switzerland) during which she tackled the design and development of a mechatronic system for the restoration of sensory-feedback in lower-limb amputees and diabetic patients. After completing her thesis, she continued working in the laboratory for 2 months as Research Assistant further developing the technology.

During her Master's degree, Chiara worked in the Implantable Bionics Laboratory at the Graduate School of Biomedical Engineering (UNSW Sydney, Australia) as part of her student exchange program. During these months, she focused on the design of a multi-channel constant current biphasic simulator for dissolution of platinum electrodes for cochlear implants. Chiara joined the Sensory-Motor Systems Laboratory (ETH Zürich, Switzerland) as a doctoral student in January 2021. Her research focuses on the development of a pediatric lightweight wearable exosuit to assist children and adolescents with neurological walking impairments across ambulatory activities of daily living. In 2021, Chiara joined the CYBATHLON team as Head of Discipline in the leg prosthesis field, supporting the organization and conduct of national and international competitions that encourage the development of assistive technologies suitable for everyday use with and for people with disabilities.

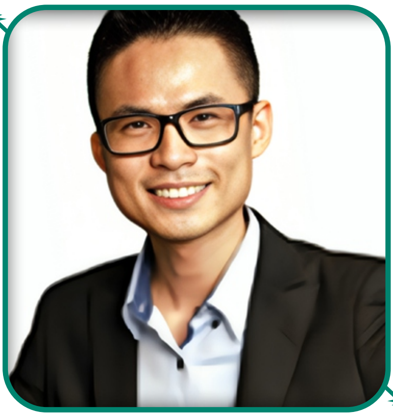

DR. KHOR KANG XIANG

Founder, and Rehab Technology Specialist at Techcare Innovation

Dr. Khor Kang Xiang graduated his Ph.D. in rehabilitation engineering and he is the co-founder, and rehab technology specialist at Techcare Innovation. Driven by a passion to assist the needy since 2012, he initiated several projects and transformed them into products that enhance people's lives. His dedication to utilizing technology for practical rehabilitation solutions has been a driving force behind Techcare Innovation. To date, he has garnered over 30 international and national innovation awards and successfully sold the products to countries in Asia and Europe, including Malaysia, Thailand, China, India, and the United Kingdom.

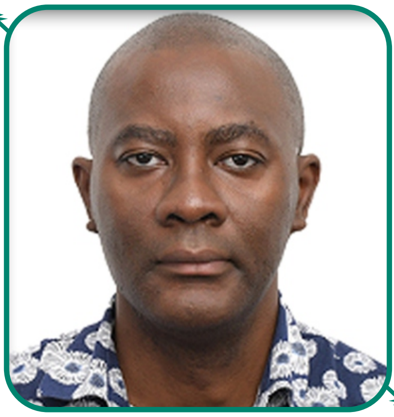

DR. LESLIE AJAVON

African fellow of the International Rehabilitation Forum (IRF) Member, World Rehabilitation Alliance (WHO) Primary Care Work-stream group advocating for the integration of Rehabilitation services into primary care. Dr. Leslie Ajavon's, is a dedicated Rehabilitation Medicine (SERM) Fellow -In-Training with Ghana College of Physician & Surgeons. He is a Family Physician at 37 Military Hospital and Sports, Exercise and member of both the West African College of Physicians and Ghana College of Physicians and Surgeons.

Dr. Ajavon's passion lies in optimizing the quality of life and function of his patients undergoing rehabilitation. His practice incorporates family medicine principles with rehabilitation strategies, allowing him to address a wide spectrum of medical conditions and functional needs of patients. He is particularly adept at managing chronic illnesses, focusing on preventive care, and tailoring rehabilitation plans to suit individual patient needs. He is currently an African fellow of the International Rehabilitation Forum (IRF) and a member of the World Rehabilitation Alliance (WHO) Primary Care Work-stream group advocating for the integration of Rehabilitation services into primary care.

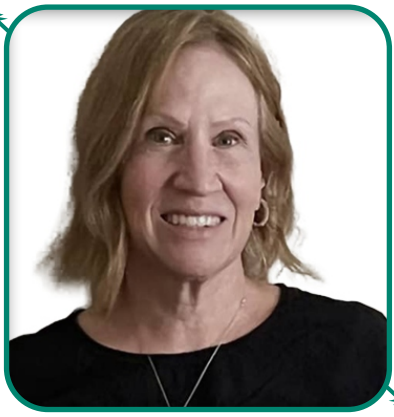

MS. MARY COLLIER BARNES

MOT, CHT, CIDN

Mary Barnes has been a CHT for over 30 years. She has been an instructor for ASHT's Education on Tour, Hands on Orthotics, the Tennessee Occupational Therapy Association, Integrative Dry Needling, the Arthritis Foundation and Belmont University. She also teaches internationally through ASHT and other organizations. She is the author and instructor of the Foundations Course and International Traveling Course.

Mary has completed international medical mission trips with Healthcare Volunteers Overseas and SurgiCorps International. She is the recipient of the AAHS International Volunteerism Award for an international mission to Ghana.

She served in the Education Division as project for lead for Education on Tour and on ASHT's International Committee. She is the former Chair of the Tennessee Board of Occupational and Physical Therapy Examiners. Mary is the recipient of many awards including TnOTA Therapist of the Year, ASHT Grassroots Award and outstanding committee.

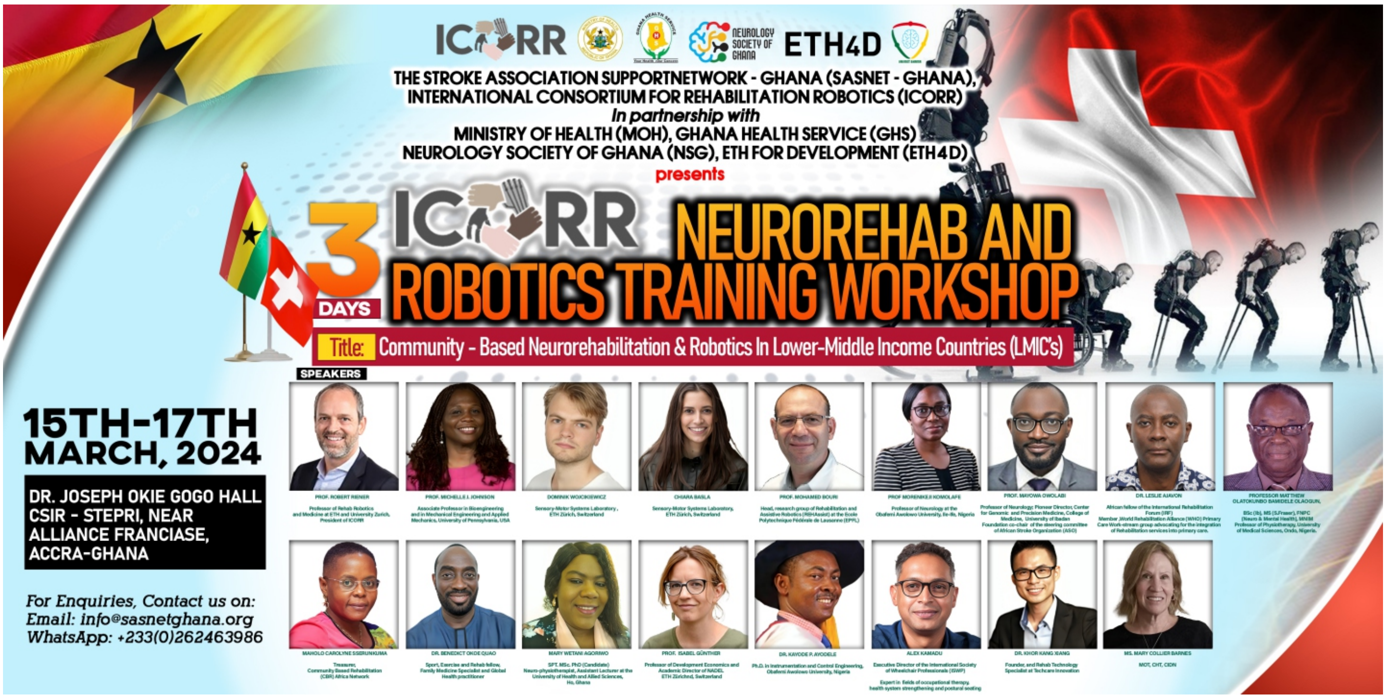

DAY 1: FRIDAY 15TH MARCH, 2024		
**TIME**	**ACTIVITY**	**FACILITATOR**
8:00AM - 9:00AM	ARRIVAL AND REGISTRATION	
**Openning Ceremony**		
9:00AM - 9:10AM	Welcome Address	Chief Dr. Ben S. Jabuni / Ad Adams Ebenezer, President / Executive Director SASNET – GHANA
9:10AM - 9:20AM	Openning Remarks	Prof. Robert Riener, President ICORR
9:20AM - 9:35AM	Remarks by Partners	Prof. Albert Akpalu, President Neurology Society of Ghana
		Ambassador Simone Giger Ambassador of Switzerland to Ghana
		Mr. Labram M.Musah National Coordinator, Ghana NCD Alliance
9:35AM - 9:50AM	Address by Ministry of Health, Ghana	Hon. Kwaku Agyeman-Manu
		Health Minister Designate Hon. Dr. Bernard Okoe Boye
9:50AM - 9:55AM	Programme Orientation	Organizing Committee
9:55AM - 10:15AM	TEN – Winneba Collaboration Cross Cultural Alternative Rehabilitation Solutions	Mr. Emmanuel Aggrey Nelson Trauma and Specialist Hospital, Winneba
10:15AM- 10:30AM	**TEA BREAK**	
**SESSION I: Moderator**		

10:30AM - 11:00AM	Promoting Brain Health in Africa Through Neurorehabilitation, New Frontier	Prof. Mayowa O. Owolabi
**Interactive session: Q&A - Moderator**		
11:00AM - 11:30AM	Clinical Situation on Assistive Device in Ghana	Dr. Leslie Ajavon
**Interactive session: Q&A - Moderator**		
11:30AM - 11:40AM	Interactive session/Break	
**11:40AM - 12:10PM**	**LUNCH BREAK**	
12:10PM - 12:45PM	Wheelchair and Mobility	Dr. Benedict Okoe Quao
**Interactive session: Q&A - Moderator**		
12:45PM - 1:20PM	Towards Inclusive Rehab & Robotics	Prof. Michelle J. Johnson
1:20PM - 1:50PM	Community Based Rehab WHO Model	Ms. Maholo Carolyne Ssenunkuma
1:50PM – 2:20PM	Therapist Management of Wrist Injuries	Ms. Mary Collier Barnes MOT, CHT, CIDN
2:20PM – 2:50PM	From Users Driven Wheelchairs to Understanding Users' Intention in Autonomous Wheelchair	Mr. Dominik Woicikiewicz
2:50PM – 3:20PM	Portable Hand Robotics Training Approach for Stroke Rehab	Dr. Khor Kang Xiang
3:20PM – 3:30PM	**Interactive session: Q&A - Moderator**	
3:30PM – 4:00PM	Wheelchair Mobility Provision – Unlocking Potentials with ISWP Resources	Mr. Alexander Komadu
4:00PM – 4:30PM	**Interactive session: Q&A - Moderator**	
4:30PM – 5:00PM	Wrapping Up & Closing	Moderator
**VENUE 2: CSIR – WATER RESOURCE STAFF CONFERENCE HALL<br>DAY 2: SATURDAY 16TH MARCH, 2024**		
**SESSION II: Moderator**		
8:00AM - 8:30AM	Recap of Day 1 Talk	Participant/Moderator

8:30AM – 9:15AM	Trends in Upper Extremity Rehab and Robotics	Prof. Robert Riener
9:15AM - 9:45AM	ETH Research & Teaching Partnership in Africa	Prof. Isabel Gunther
9:45AM – 10:05AM	**Interactive Session & Break: Q&A - Moderator**	
10:05AM - 10:35AM	Lower Extremity Exoskeleton & Exosuit for Children and Adult	Ms. Chiara Basla
10:35AM - 11:05AM	Rehabilitation in Post - Stroke Management	Mrs. Mary Agoriwo
11:05AM – 11:15AM	Interactive Session: Q&A- Moderator	
11:15AM – 11:45AM	Mechatronics Solution for Lower Limbs, Rehab, Mobility, Evaluate and Interact	Prof. Mohamad Bouri
11:45AM – 12:15PM	Robotics in Clinical Practice	Professor Matthew Olatokunbo Bamidele Olaogun<br>Prof. M. A. Komolafe<br>Dr. Kayode P. Ayodele
12:15PM – 12:35PM	Interactive Session & Break: Q&A- Moderator	
12:35PM – 1:05PM	Wrap Up & Recap of All Sessions	Moderator
1:05PM – 1:15PM	Announcement of Certificates and Closing Ceremony	Organizing Team
1:15PM – 1:20PM	Network Dinner	All

NOTES

ICORR

MINISTRY OF HEALTH
REPUBLIC OF GHANA

GHANA HEALTH SERVICE
GHS
Your Health • Our Concern

NEUROLOGY
SOCIETY OF
GHANA

ETH4D
ETH FOR DEVELOPMENT

SASNET GHANA

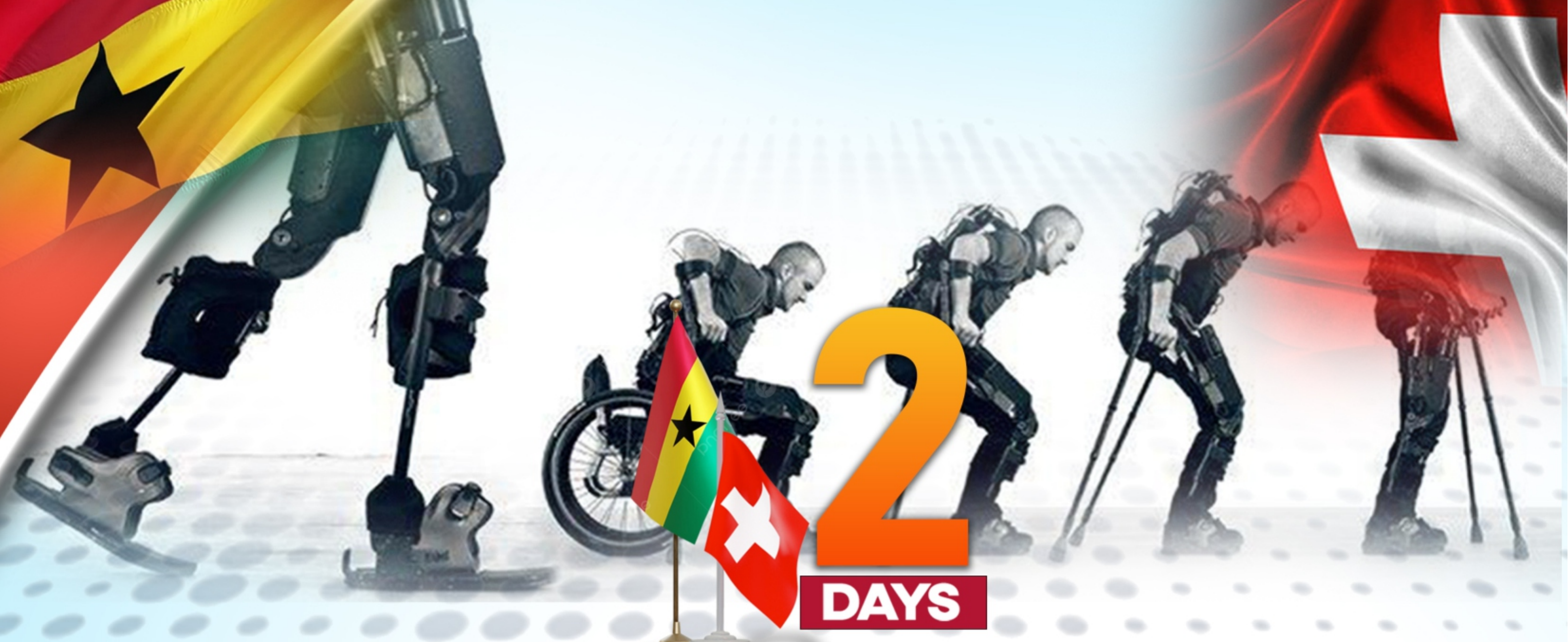

2 DAYS

ICORR

NEUROREHAB AND ROBOTICS TRAINING WORKSHOP

Title: Community - Based Neurorehabilitation & Robotics In Lower-Middle Income Countries (LMIC's)

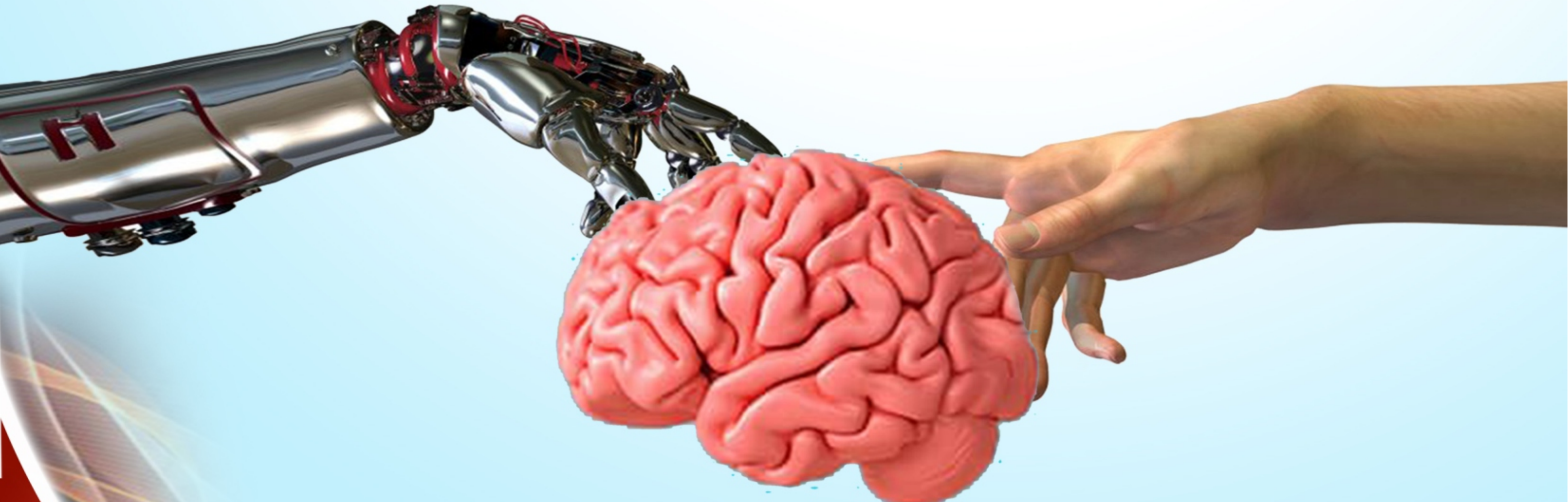

For Enquiries, Contact us on:
Email: info@sasnetghana.org
WhatsApp: +233(0)262463986
