# Supplementary material for: Pragmatic recommendations to improve access to rehabilitation robots, assistive technologies and neurorehabilitation services in Africa: proceedings from ICORR-SASNET Ghana neurorehabilitation workshop, 2024
Source: Front Stroke. 2025 Sep 1;4:1565651. doi: 10.3389/fstro.2025.1565651 (PMC12802663; doi:10.3389/fstro.2025.1565651)
Supplement: Supplementary file 4 [file Supplementary_file_4.pdf]

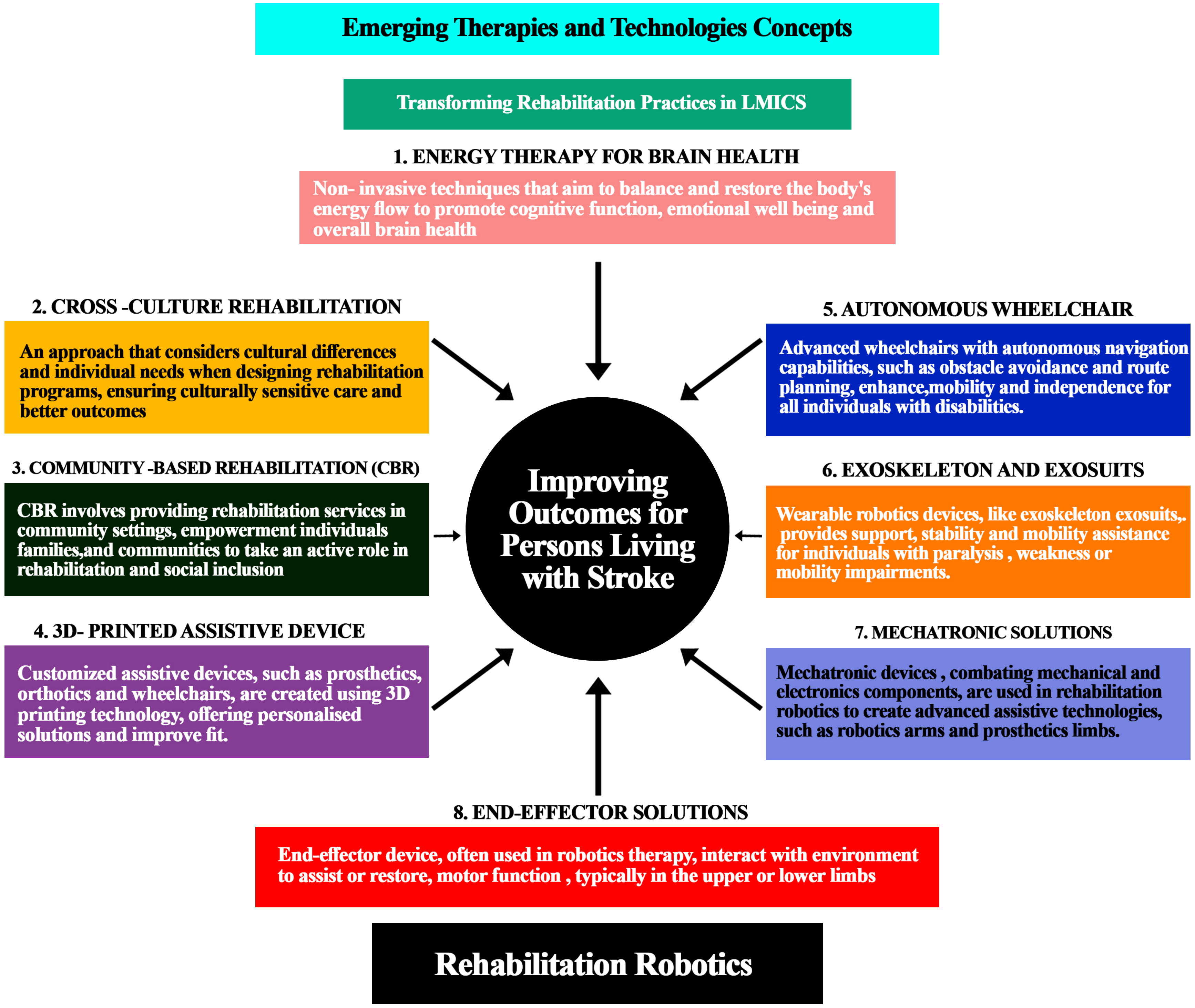
Emerging Therapies and Technologies Concepts
Transforming Rehabilitation Practices in LMICS
1. ENERGY THERAPY FOR BRAIN HEALTH
Non- invasive techniques that aim to balance and restore the body's energy flow to promote cognitive function, emotional well being and overall brain health
2. CROSS -CULTURE REHABILITATION
An approach that considers cultural differences and individual needs when designing rehabilitation programs, ensuring culturally sensitive care and better outcomes
3. COMMUNITY -BASED REHABILITATION (CBR)
CBR involves providing rehabilitation services in community settings, empowerment individuals families,and communities to take an active role in rehabilitation and social inclusion
4. 3D- PRINTED ASSISTIVE DEVICE
Customized assistive devices, such as prosthetics, orthotics and wheelchairs, are created using 3D printing technology, offering personalised solutions and improve fit.
Improving Outcomes for Persons Living with Stroke
5. AUTONOMOUS WHEELCHAIR
Advanced wheelchairs with autonomous navigation capabilities, such as obstacle avoidance and route planning, enhance,mobility and independence for all individuals with disabilities.
6. EXOSKELETON AND EXOSUITS
Wearable robotics devices, like exoskeleton exosuits,. provides support, stability and mobility assistance for individuals with paralysis , weakness or mobility impairments.
7. MECHATRONIC SOLUTIONS
Mechatronic devices , combating mechanical and electronics components, are used in rehabilitation robotics to create advanced assistive technologies, such as robotics arms and prosthetics limbs.
8. END-EFFECTOR SOLUTIONS
End-effector device, often used in robotics therapy, interact with environment to assist or restore, motor function , typically in the upper or lower limbs
Rehabilitation Robotics
